# PARP1 deficiency mitigates amyloid pathology, neurodegeneration, and cognitive decline in a familial Alzheimer’s disease model

**DOI:** 10.1073/pnas.2525028123

**Published:** 2026-05-15

**Authors:** Aanishaa Jhaldiyal, Manisha Kumari, Lauren C. Guttman, Trupti Tripathi, Mohammed Repon Khan, Justin Wang, Devanik Biswas, Abhishek Pasupuleti, Akansha Aggarwal, Shraddha Pandya, Shih-Ching Chou, Nikhil Panicker, Abhay Monghekar, Marilyn Albert, Lynn M. Bekris, James B. Leverenz, Tae-In Kam, Ted M. Dawson, Valina L. Dawson

**Affiliations:** ^a^Neuroregeneration and Stem Cell Programs, Institute for Cell Engineering, The Johns Hopkins University School of Medicine, Baltimore, MD 21205; ^b^Department of Neurology, The Johns Hopkins University School of Medicine, Baltimore, MD 21205; ^c^Department of Physiology, Pharmacology and Therapeutics, The Johns Hopkins University School of Medicine, Baltimore, MD 21205; ^d^Department of Biochemistry and Molecular Biology, Bloomberg School of Public Health, Johns Hopkins University School of Medicine, Baltimore, MD 21205; ^e^ Department of Laboratory Medicine and Pathology, University of Washington, Seattle, WA 98104; ^f^ Lerner Research Institute, Genomic Medicine, Cleveland Clinic, Cleveland, OH 44195; ^g^Lou Ruvo Center for Brain Health, Neurological Institute, Cleveland Clinic, Cleveland, OH 44195; ^h^ Department of Neurology, Cleveland Clinic, Cleveland, OH 44195; ^i^ Geriatric Research Education and Clinical Center, Veteran Affairs Puget Sound Health Care System (S-182), Seattle, WA 98108; ^j^ Department of Neurology, University of Washington, Seattle, WA 98195; ^k^Solomon H. Snyder Department of Neuroscience, The Johns Hopkins University School of Medicine, Baltimore, MD 21205

**Keywords:** poly(ADP-ribose) (PAR), PARP, Alzheimer’s disease, amyloid beta (Aβ), neurodegeneration

## Abstract

Poly(ADP-ribose) (PAR) polymerase 1 (PARP1) has been implicated in DNA damage responses and neuroinflammation in Alzheimer’s disease (AD), yet its role in amyloid-beta (Aβ) pathology remains unclear. Here, we show that PARP1 activation drives Aβ pathology and neurodegeneration. Using a sensitive enzyme-linked immunosorbent assay, we observed significantly elevated PAR levels in the cerebrospinal fluid (CSF) of patients with mild cognitive impairment (MCI) and AD compared to controls. In vitro, oligomeric Aβ_1-42_ activated PARP1 and induced DNA damage, while genetic or pharmacological inhibition of PARP1 conferred neuroprotection. In vivo, PARP1 knockout in the 5XFAD mouse model of amyloidosis led to reduced amyloid plaque burden, preserved synaptic and neuronal integrity, attenuated glial activation and neuroinflammation, and rescued cognitive deficits. Mechanistically, PARP1 deficiency decreased amyloid precursor protein and BACE1 levels, altered γ-secretase complex composition, and enhanced Aβ degradation via neprilysin. These findings position PARP1 as a critical mediator of Aβ toxicity and neurodegeneration, suggesting its inhibition as a promising therapeutic strategy for AD.

## Significance

Our study identifies poly(ADP-ribose) (PAR) as an elevated biomarker in the cerebrospinal fluid of patients with mild cognitive impairment and Alzheimer’s disease, correlating with established markers of amyloid pathology. We demonstrate that PARP1, the enzyme responsible for PAR synthesis, is activated by neurotoxic Aβ_1-42_ and mediates neuronal death, amyloid plaque formation, neuroinflammation, and cognitive deficits in a mouse model of Alzheimer’s disease. Importantly, genetic ablation of PARP1 not only protects neurons from Aβ toxicity but also reduces amyloid burden by suppressing Aβ production and enhancing its degradation. These findings highlight PARP1 as a critical regulator of amyloid pathology and neurodegeneration, and suggest that PARP1 inhibition may offer a promising therapeutic avenue for Alzheimer’s disease by simultaneously targeting multiple pathogenic mechanisms.

Poly (ADP-ribose) polymerase 1 (PARP1) activation leads to neuronal cell death following glutamate excitotoxicity and stroke (1–4), and neurodegeneration in Parkinson’s disease (PD) (5–7). This form of cell death is designated parthanatos, and is distinct from other types of cell death such as apoptosis and necrosis (8, 9). Interference of each step in the parthanatic cascade has conferred neuroprotection in a variety of models (10–12). It has generally been thought that PARP1 activity and parthanatos primarily play a role in acute cellular injury such as stroke and/or glutamate excitotoxicity. The view was changed with our recent report that PARP1 activity and parthanatos contribute to neurodegeneration in a chronic animal model of PD in which we found that degeneration of dopamine (DA) neurons in the substantia nigra pars compacta (SNpc) in the pathologic α-synuclein model of PD generated by intrastriatal injections of α-synuclein preformed fibrils (α-syn PFF) was primarily driven by PARP1 activity and parthanatos (5, 13, 14). Activation of macrophage migration inhibitory factor (MIF) nuclease activity, the final executioner of parthanatos, drives pathology in three orthogonal models of PD further implicating parthanatos in a chronic neurodegenerative disorder (14). In postmortem human Alzheimer’s Dementia (AD) brain, there is evidence of elevated PARP1 activity where Love et al. reported that product of PARP1 activity, Poly (ADP-ribose) (PAR) was elevated in the frontal and temporal cortices of AD patients vs. controls (15). PARP1 polymorphisms may also decrease the risk and severity of AD (16).

However, the precise role of parthanatos in driving neuronal loss in AD is unknown. There are several transgenic models of AD (17) The 5XFAD transgenic mouse model has been extensively used to study amyloid beta (Aβ) pathology in mice. The 5XFAD transgenic mice coexpress a total of five FAD mutations [APP K670N/M671L (Swedish) + I716V (Florida) + V717I (London) and PS1 M146L+ L286V] (18). In addition to exhibiting memory impairment the 5XFAD transgenic mouse is one of the few Aβ animal models that exhibits significant neuron loss (19). By 9 mo of age 5XFAD mice exhibit visible loss of large pyramidal neurons in cortical Layer 5 and the subiculum. Thus, 5XFAD mice allow for the investigation of whether parthanatos contributes to driving neuronal loss in the context of pathologic Aβ.

We crossed the 5XFAD mice with PARP1^−/−^ mice and report that the absence of PARP1 significantly reduces the neuronal loss, Aβ plaque load, and cognitive deficits in the 5XFAD mice. This decrease in pathology was also accompanied by changes in markers of neuroinflammation and attenuation of glial pathology. Moreover, we reveal PARP1’s involvement in altering amyloid precursor protein (APP) metabolism, suggesting the enzyme plays a broader role in the pathogenesis of AD beyond its influence on neuronal loss.

## Results

### Increased Levels of PAR in the Cerebrospinal Fluid (CSF) of Patients with Mild Cognitive Impairment (MCI) and AD.

**Fig. 1.**
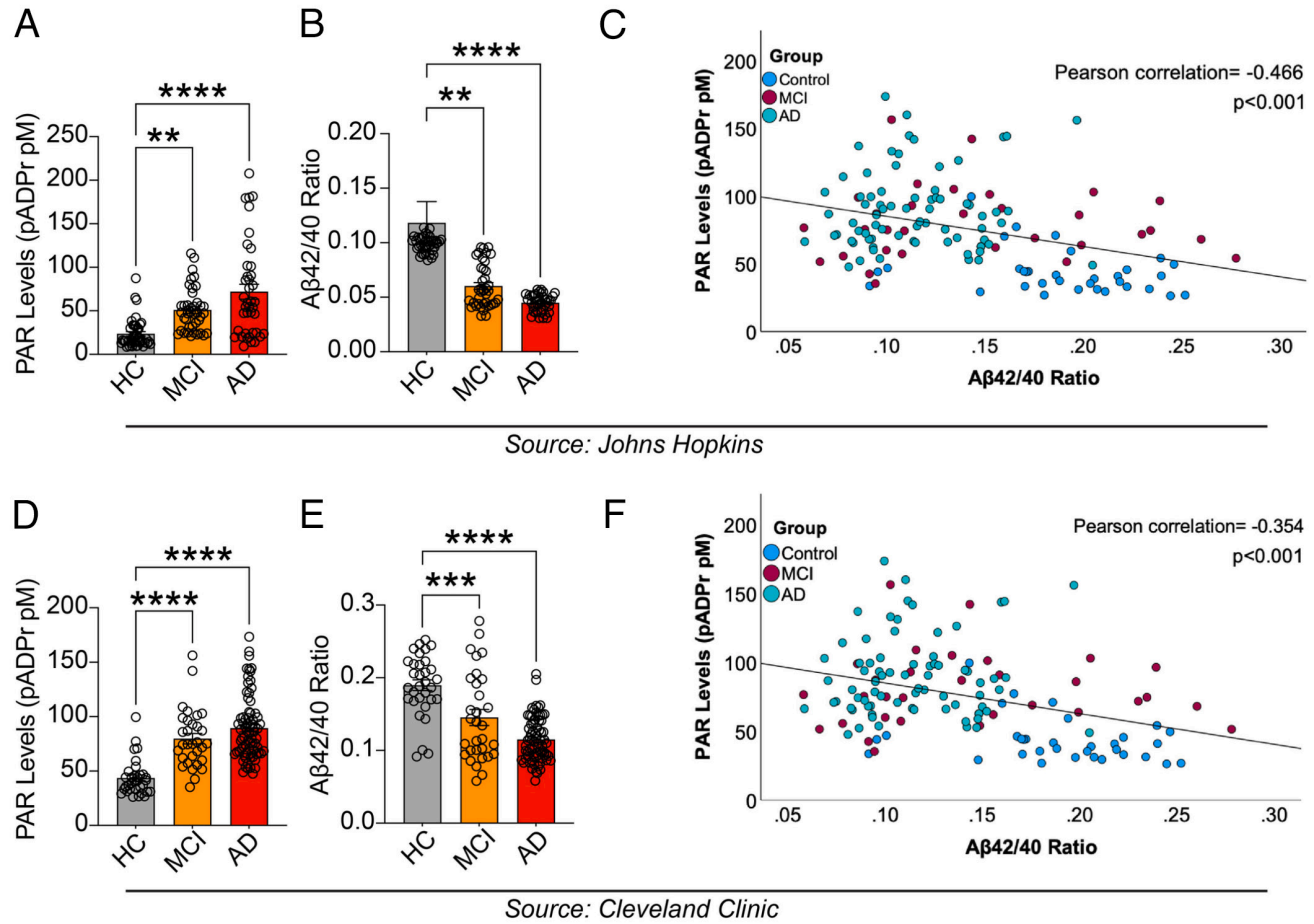
PAR levels are increased in patients with mild cognitive deficit (MCI) and AD. (*A*) PAR levels from CSF samples from Johns Hopkins Medicine of patients with MCI (n = 40) and AD (n = 40) vs. HC (n = 40) as determined by PAR ELISA. Bars represent means ± SEM. One-way ANOVA was followed by Tukey’s multiple comparisons test: HC vs. MCI, *P* = 0.0025; HC vs. AD, *P* < 0.0001; MCI vs. AD, *P* = 0.0277. (*B*) Aβ42/40 ratio with advancing stages of AD (HC>MCI>AD) of CSF samples from Johns Hopkins Medicine. Bars represent means ± SEM. One-way ANOVA was followed by Tukey’s multiple comparisons test: HC vs. MCI, *P* = 0.0013; HC vs. AD, *P* < 0.0001; MCI vs. AD, *P* = 0.6085 (ns). (*C*) Scatter plot of the correlation between CSF PAR (from Johns Hopkins Medicine) with Aβ40/42 ratio in HC, MCI, and AD. Correlation coefficients (r) and *P*-values were calculated using Pearson’s correlation analysis across all CSF samples, including HC, MCI, and AD groups. (*D*) PAR levels in CSF samples from Cleveland Clinic of patients with MCI (n = 31) and patients with AD (n = 74) vs. HCs (n = 33) as determined by PAR ELISA. Bars represent means ± SEM. One-way ANOVA was followed by Tukey’s post hoc test. One-way ANOVA was followed by Tukey’s multiple comparisons test: HC vs. MCI, *P* < 0.0001; HC vs. AD, *P* < 0.0001; MCI vs. AD, *P* = 0.2046 (ns). (*E*) Aβ42/40 ratio of CSF samples from Cleveland Clinic in advancing stages of AD (HC>MCI>AD). Bars represent means ± SEM. One-way ANOVA was followed by Tukey’s post hoc test. One-way ANOVA was followed by Tukey’s multiple comparisons test: HC vs. MCI, *P* = 0.0001; HC vs. AD, *P* < 0.0001; MCI vs. AD, *P* = 0.0026. (*F*) Scatter plot of the correlation between CSF PAR (from Cleveland Clinic) with Aβ40/42 ratio in HC, MCI, and AD. Correlation coefficients (r) and *P*-values are from Pearson’s correlation analysis was calculated using all CSF samples (HC, MCI, and AD).

A sensitive enzyme-linked immunosorbent assay (ELISA) for PAR (5) was used to determine whether PAR is elevated in the CSF of patients with MCI or AD compared to controls. PAR levels were significantly elevated in patients with MCI and AD vs. healthy controls (HC) in two independent patient cohorts (Fig. 1 *A* and *D* and Dataset S1). The Aβ42/40 ratio progressively decreases along the Alzheimer’s continuum from MCI to dementia (20). Accordingly, we observed the same trend in our CSF samples (Fig. 1 *B* and *E*). We also performed Pearson correlation analysis to examine the relationship between Aβ42/40 and PAR across HC, MCI, and AD samples, representing the disease continuum. In both cohorts, we found a negative correlation between PAR levels and the Aβ42/40 ratio in MCI samples (Fig. 1 *C* and *F*). Collectively, these data revealed increased CSF PAR in both MCI and AD, suggesting a potential role for PAR and PARP1 in AD pathogenesis.

### Oligomeric Aβ_1-42_ Activates PARP1, and Genetic Deletion and Pharmacological Inhibition of PARP1 Protects against Aβ_1-42_ Neurotoxicity.

**Fig. 2.**
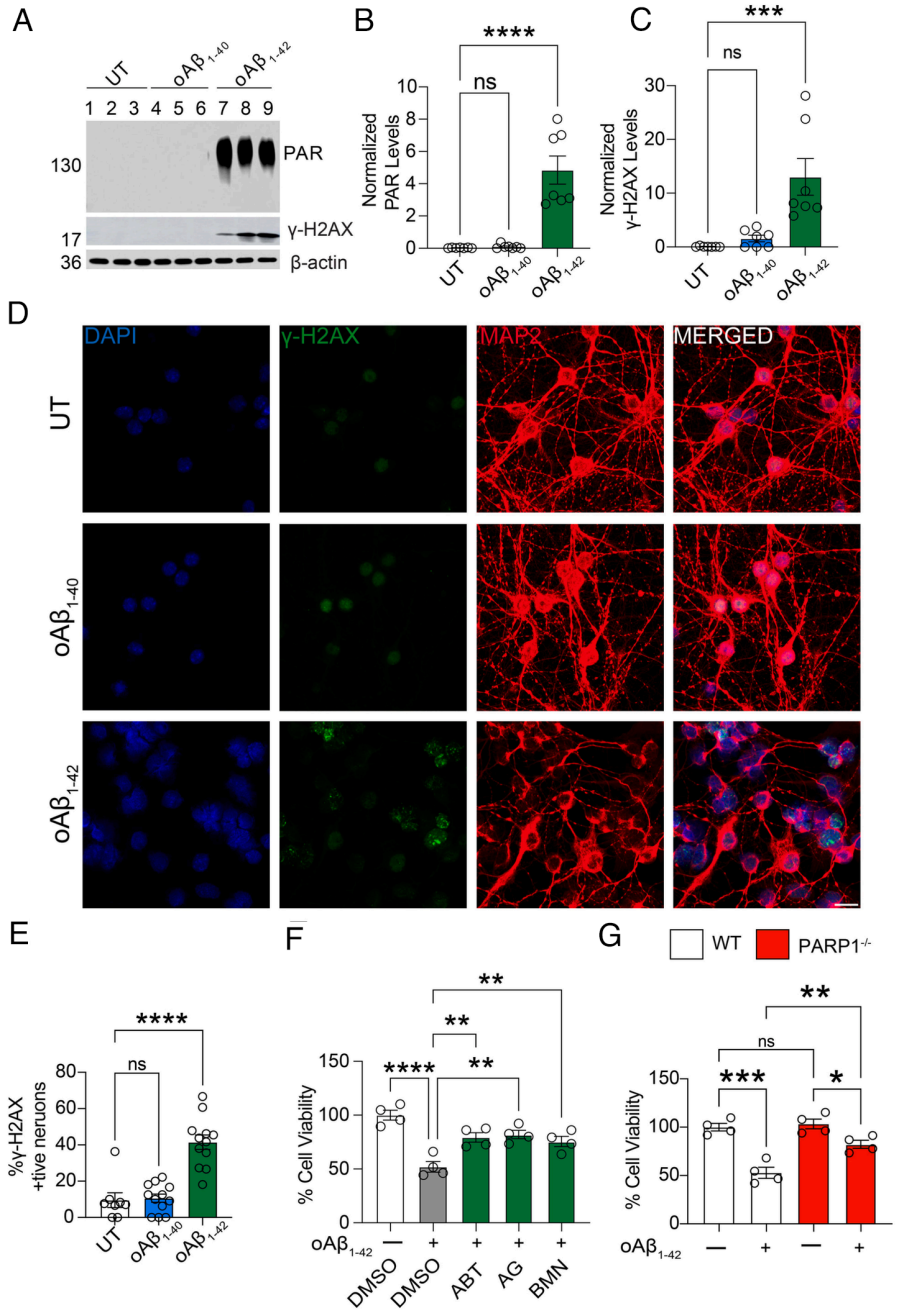
Oligomeric Aβ_1-42_ (oAβ_1-42_) treatment causes PAR activation in primary cortical neurons. (*A*–*C*) Representative (*A*) immunoblot and quantification of (*B*) PAR and (*C*) γ-H2AX levels in primary cortical neurons UT or treated with 1 µM oAβ_1-40_ or oAβ_1-42_ for 24 h. Bars represent means ± SEM (n = 6-7). One-way ANOVA was followed by post hoc multiple comparisons testing against the untreated (UT) control. PAR (*B*): UT vs. oAβ1–40, *P* = 0.9930 (ns); UT vs. oAβ1–42, *P* < 0.0001. γ-H2AX (*C*): UT vs. oAβ1–40, *P* = 0.8155 (ns); UT vs. oAβ1–42, *P* = 0.0005. (*D* and *E*) γ-H2AX–positive nuclei treated with 1 µM oAβ_1-42_ or 1 µM oAβ_1-40_ or in UT controls. (*D*) Representative γ-H2AX immunofluorescence. (Scale bar, 20 µm.) (*E*) Quantification of γ-H2AX–positive nuclei in UT, 1 µM oAβ_1-40,_ and 1 µM oAβ_1-42_ cultures. Bars represent mean ± SEM (n = 12). One-way ANOVA was followed by post hoc multiple-comparisons testing against the UT control: UT vs. oAβ1–40, *P* = 0.9651 (ns); UT vs. oAβ1–42, *P* < 0.0001. (*F*) Alamar blue cell viability assay from primary cortical neurons preincubated with 1 µM of ABT-888, AG-014699, or BMN 673 for 1 h and further incubated with 1 µM oAβ_1-42_ for 2 d. Bars represent mean ± SEM (n = 4). Two-way ANOVA was followed by post hoc multiple-comparisons testing against the oAβ1–42 (Aβ) condition: Aβ vs. DMSO, *P* < 0.0001; Aβ vs. Aβ + ABT-888, *P* = 0.0025; Aβ vs. Aβ + AG-014699, *P* = 0.0011; Aβ vs. Aβ + BMN 673, *P* = 0.0079. (*G*) Alamar blue cell viability assay from WT or PARP1^-/-^ primary cortical neurons and further incubated with 1 µM oAβ_1-42_ for 2 d. Bars represent mean ± SEM (n = 4). Two-way ANOVA was followed by post hoc multiple-comparisons testing: WT vs. WT + oAβ1–42, *P* = 0.0002; PARP1^-/-^ vs. PARP1^-/-^ + oAβ1–42, *P* = 0.0349; WT vs. PARP1^-/-^, *P* = 0.9762 (ns); WT + oAβ1–42 vs. PARP1^-/-^ + oAβ1–42, *P* = 0.0046.

To explore a link between AD pathogenesis and PARP1, oligomeric Aβ species (*SI Appendix*, Fig. S1*A*) were added to primary cortical neuron cultures to determine if PARP1 becomes activated. Either Aβ_1-40_ (1 µM) or Aβ_1-42_ (1 µM) was applied to primary cortical neurons and PARP1 activity was assessed 24 h later via PAR immunoblot analysis (Fig. 2 *A* and *B*). Aβ_1-42_ administration led to a significant increase in PAR immunoreactivity while Aβ_1-40_ had no effect (Fig. 2 *A* and *B*). We also evaluated the presence of γ-H2AX, a marker of DNA damage, which is concurrently elevated with PARP1 activity (Fig. 2 *A* and *C*) (21). Accompanying the increase in PAR levels was a statistically significant increase in γ-H2AX (Fig. 2 *A* and *C*). γ-H2AX immunoreactivity was also significantly increased in the nuclei of cortical neurons treated with Aβ_1-42_ for 24 h (Fig. 2 *D* and *E*). Oligomeric Aβ_1-42_ (1 µM) administration significantly increased PAR levels as early as 8 h, and there were further significant increases at 24 h and 48 h (*SI Appendix*, Fig. S1 *B* and *C*). A significant and steady increase in γ-H2AX was also observed along these time points (*SI Appendix*, Fig. S1 *B* and *D*). These results indicate that oligomeric Aβ_1-42_ damages DNA and is an activator of PARP1.

Oligomeric Aβ_1–42_ increased cellular PAR levels in primary cortical neurons, and pretreatment with the PARP inhibitors: ABT-888 (veliparib), AG-014699 (rucaparib), or BMN 673 (talazoparib) (1 µM) significantly reduced Aβ-induced PAR accumulation (*SI Appendix*, Fig. S1 *E* and *F*). We next asked whether suppressing this PARP activation translated into improved neuronal survival. In the Alamar Blue viability assay, oligomeric Aβ_1–42_ toxicity was significantly reduced in wild type (WT) neurons treated with ABT-888, AG-014699, or BMN 673 (Fig. 2*F*). In addition, ABT-888 (1 µM) significantly reduced oligomeric Aβ_1–42_ induced cell death as assessed by propidium iodide (PI) staining (*SI Appendix*, Fig. S1 *G* and *H*). PARP1^−/−^ neurons were also significantly resistant to oligomeric Aβ_1–42_ neurotoxicity compared to WT neurons (Fig. 2*G*), and oligomeric Aβ_1–42_ failed to induce PAR in PARP1^−/−^ neurons (*SI Appendix*, Fig. S1*I*). Together, these findings demonstrate that PARP1 activation contributes to oligomeric Aβ_1-42_ - induced neurotoxicity, and that both pharmacological inhibition and genetic ablation of PARP1 confer significant neuroprotection.

### The Absence of PARP1 Reduces the Aβ Plaque Load and Prevents Neuronal Cell Death in 5XFAD Mice.

The role of PARP1 in Aβ pathology, neurobehavior, and neurodegeneration was assessed using 5XFAD transgenic mice (18). 5XFAD mice were crossed to PARP1^−/−^ mice to generate 5XFAD/PARP1^+/−^ mice, which were then crossed with PARP1^+/−^ mice. Approximately 20 male and female mice of WT, 5XFAD, PARP1^−/−^, and 5XFAD/PARP1^−/−^ were generated and aged to 9 mo (*SI Appendix*, Fig. S2*A*). Agarose gel and immunoblot analysis confirmed the absence of PARP1 in 5XFAD/ PARP1^−/−^ and PARP1^−/−^ mice (*SI Appendix*, Fig. S2 *B* and *C*). APP mRNA level as determined by RT-PCR was significantly upregulated in 5XFAD mice compared to WT and PARP1^−/−^ mice. There was no significant difference in the APP mRNA level in 5XFAD vs. 5XFAD/ PARP1^−/−^ mice (*SI Appendix*, Fig. S2*D*).

**Fig. 3.**
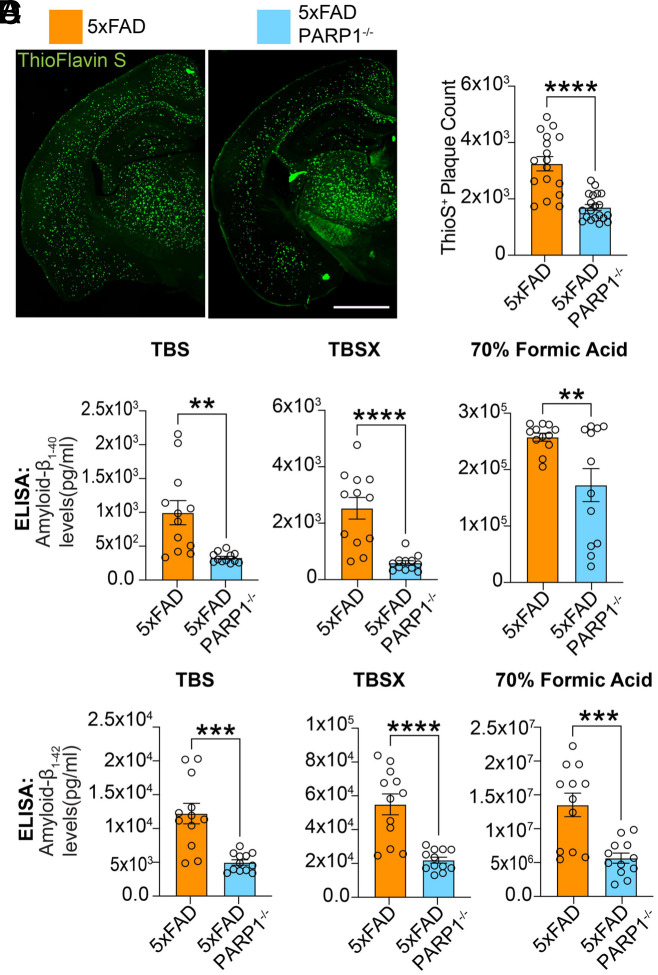
PARP1 deletion decreases Aβ plaque neuropathology in 5XFAD mice. (*A*) Immunofluorescence images and (*B*) quantification of Thioflavin S staining of amyloid-β plaques in 5XTg FAD and 5XFAD/PARP1^−/−^ mice. (Scale bar 1,000 µm.) Bars represent mean ± SEM (n = 10). Unpaired two-tailed Student’s *t* test: *P* < 0.0001 (t = 5.987, df = 35). (*C* and *D*) ELISA quantification of (*C*) Aβ_1–40_ and (*D*) Aβ_1–42_ levels in cortical lysates from 5xFAD and 5xFAD/PARP1^−/−^ mice across sequential extraction fractions (TBS, TBSX, and 70% formic acid). Bars represent mean ± SEM (n = 12). Unpaired two-tailed Student’s *t* tests were performed for each fraction: Aβ_1–40_: TBS, *P* = 0.0012 (t = 3.730, df = 22); TBSX, *P* = 0.0100 (t = 2.821, df = 22); 70% FA, *P* < 0.0001 (t = 4.972, df = 22). Aβ_1–42_: TBS, *P* = 0.0001 (t = 4.658, df = 22); TBSX, *P* = 0.0004 (t = 4.154, df = 22); 70% FA, *P* < 0.0001 (t = 5.134, df = 22).

5XFAD/PARP1^−/−^ animals exhibited a ~50% reduction in Thioflavin S–positive plaque area compared with age-match littermate 5XFAD controls (Fig. 3 *A* and *B*). To assess whether this decrease in plaque burden corresponded to changes in Aβ species, human Aβ_1-40_ and Aβ_1-42_ levels were measured by ELISA. In 5XFAD/PARP1^−/−^ mice, levels of Aβ_1-40_ and Aβ_1-42_ were significantly lower in TBS-soluble, TBS + Triton X-100–soluble, and 70% formic acid–soluble fractions relative to 5XFAD mice (Fig. 3 *C* and *D*). To confirm the reduction in aggregated amyloid species, we performed immunoblot analysis of high–molecular–weight Aβ aggregates isolated by sucrose cushion ultracentrifugation. This revealed elevated levels of aggregated Aβ and PAR in 5XFAD mice compared to 5XFAD/PARP1^−/−^ and littermate controls (WT and PARP1^−/−^) mice (*SI Appendix*, Fig. S3 *A*–*C*).

**Fig. 4.**
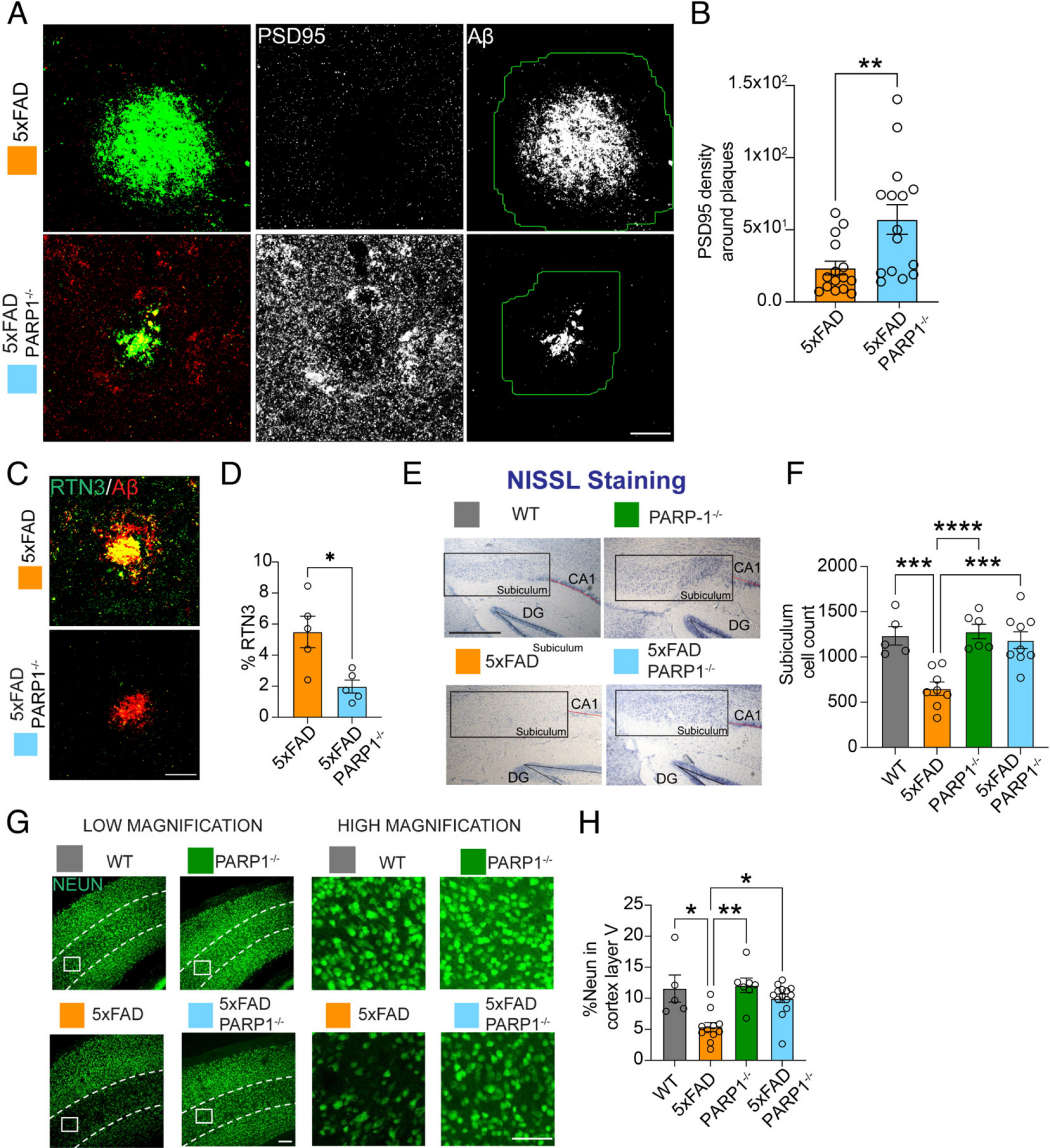
PARP1 deletion increases neuronal survival and synaptic activity in 5XFAD mice. (*A* and *B*) Immunofluorescence images and quantification of PSD95 density surrounding amyloid-β plaques in 5XFAD and 5XFAD/PARP1^−/−^ mice. Cumulative PSD95 density was measured within a 30 µm radius of each plaque. (Scale bar, 40 µm.) Bars represent mean ± SEM (n = 15). Unpaired two-tailed Student’s *t* test: *P* = 0.0059 (t = 2.981, df = 28). (*C* and *D*) Immunofluorescent images and quantification of RTN3 levels around amyloid-β plaques in 5XFAD and 5XFAD/PARP1^−/−^ mice. (Scale bar, 40 µm.) Bars represent mean ± SEM (n = 5). Unpaired two-tailed Student’s *t* test: *P* = 0.0126 (t = 3.201, df = 8). (*E* and *F*) Nissl images and quantification of WT, PARP1^−/−^, 5XFAD, and 5XFAD/PARP1^−/−^ mice. (Scale bar, 400 µm.) Bars are means ± SEM (n = 4 to 10). Two-way ANOVA was followed by post hoc multiple-comparisons testing comparing 5xFAD to the indicated genotypes: 5xFAD vs. WT, P = 0.0002; 5xFAD vs. PARP1^−/−^, *P* < 0.0001; 5xFAD vs. 5xFAD/PARP1^−/−^, P = 0.0001. (*G* and *H*) NeuN staining immunofluorescence images and quantification of cortex layer V in WT, PARP1^−/−^, 5XFAD, and 5XFAD/PARP1^−/−^ mice. Scale bar, 40 µm (Low Magnification) and 20 µm (High Magnification). Bars are means ± SEM (n = 5 to 7). Two-way ANOVA was followed by post hoc multiple-comparisons testing comparing 5xFAD to the indicated genotypes: 5xFAD vs. WT, *P* = 0.0169; 5xFAD vs. PARP1^−/−^, *P* = 0.0040; 5xFAD vs. 5xFAD/PARP1^−/−^, *P* = 0.0174.

Accompanying the reduction in Aβ core plaques was a preservation of synaptic density as assessed by PSD95 immunoreactivity density near Aβ core plaques labeled by 4G8 immunoreactivity (Fig. 4 *A* and *B*). The accumulation of Aβ into fibrillar amyloid plaques causes damage to nearby neuronal structures, leading to the development of enlarged, swollen axons and dendrites around the plaques, a phenomenon known as neuritic dystrophy. These swollen neurites contain aggregates of several proteins, such as RTN-3, which can serve as markers for this dystrophic process (22, 23). RTN-3 levels were reduced in amyloid plaque–rich regions of 5XFAD/PARP1^−/−^ mice compared with 5XFAD controls (Fig. 4 *C* and *D*).

In 5XFAD mice the subiculum and cortical layer V are areas with the most severe amyloidosis and neuron loss that begins at about 6 mo of age (18, 19). Nonbiased stereological cell counting revealed that neuronal cell loss was prevented in the 5XFAD/PARP1^−/−^ mice as assessed by Nissl stain in the subiculum of the hippocampus (Fig. 4 *E* and *F*). NeuN immunostaining intensity was significantly preserved in layer V of the cerebral cortex in 5XFAD/PARP1^−/−^ mice compared to 5XFAD mice (Fig. 4 *G* and *H*).

### Attenuation of Markers of Glial Pathology and Neuroinflammation in PARP1^−/−^ Mice.

**Fig. 5.**
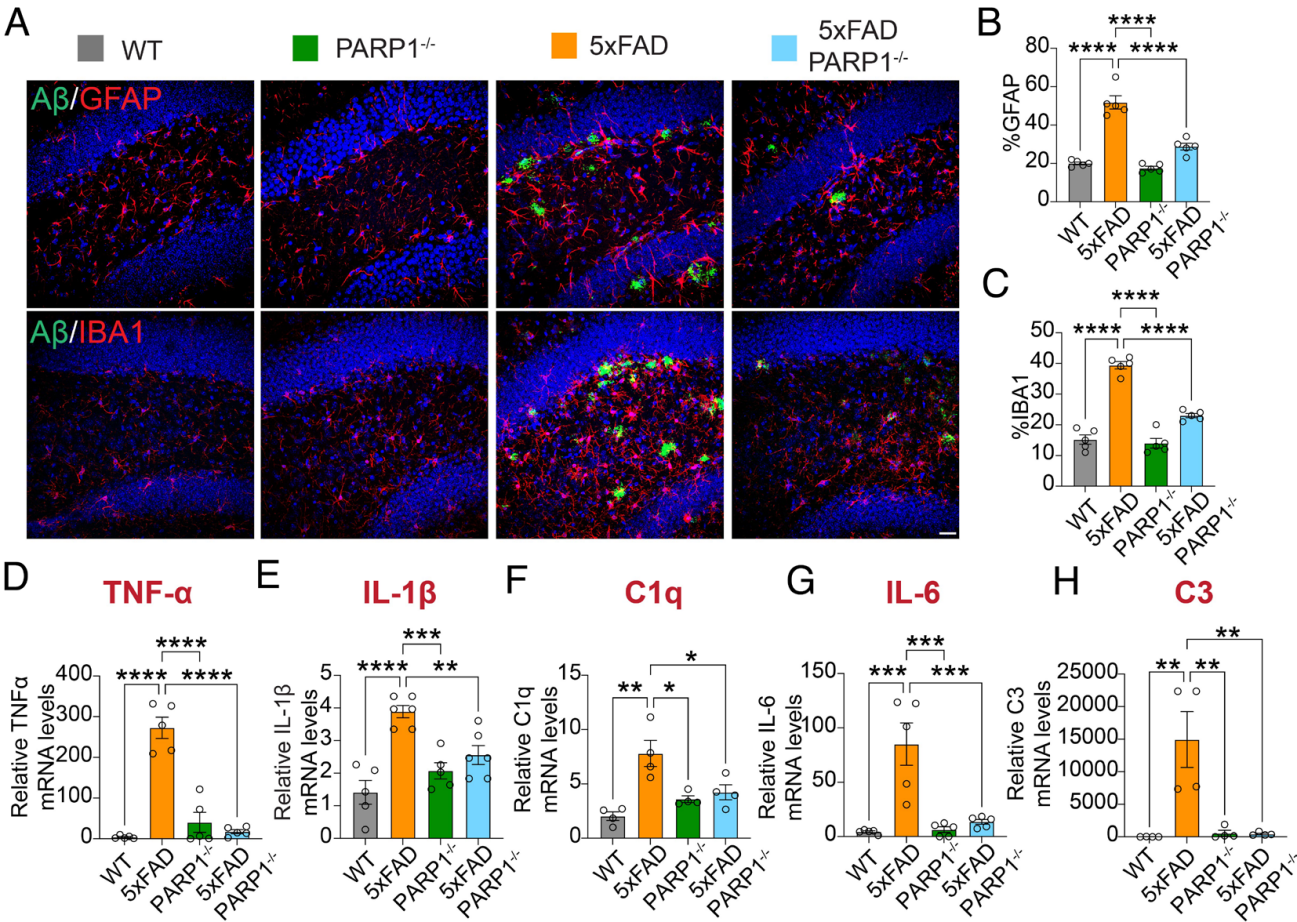
5XFAD/PARP1^−/−^ mice exhibit reduced gliosis. (*A*–*D*) Immunofluorescent images and quantification of IBA1 and GFAP levels in WT, PARP1^−/−^, 5XFAD, and 5XFAD/PARP1^−/−^. (Scale bar, 40 µm.) Bars are means ± SEM (n = 5). Two-way ANOVA was followed by post hoc multiple-comparisons testing comparing 5xFAD to the indicated genotypes. GFAP: 5xFAD vs. WT, *P* < 0.0001; 5xFAD vs. PARP1^−/−^, *P* < 0.0001; 5xFAD vs. 5xFAD/PARP1^−/−^, *P* < 0.0001. IBA1: 5xFAD vs. WT, *P* < 0.0001; 5xFAD vs. PARP1^−/−^, *P* < 0.0001; 5xFAD vs. 5xFAD/PARP1^−/−^, *P* < 0.0001. (*D*–*H*) mRNA levels of TNF-α, IL-1β, C1q, IL-6, and C3, as determined by RT-PCR from cortical lysates of WT, PARP1^−/−^, 5XFAD, and 5XFAD/PARP1^−/−^. Bars represent mean ± SEM (n = 5). Two-way ANOVA was followed by post hoc multiple-comparisons testing comparing 5xFAD to the indicated genotypes. TNF-α: 5xFAD vs. WT, *P* < 0.0001; 5xFAD vs. PARP1^−/−^, *P* < 0.0001; 5xFAD vs. 5xFAD/PARP1^−/−^, *P* < 0.0001. IL-1β: 5xFAD vs. WT, *P* < 0.0001; 5xFAD vs. PARP1^−/−^, *P* < 0.0001; 5xFAD vs. 5xFAD/PARP1^−/−^, *P* = 0.0072. C1q: 5xFAD vs. WT, *P* = 0.0025; 5xFAD vs. PARP1^−/−^, *P* = 0.0167; 5xFAD vs. 5xFAD/PARP1^−/−^, *P* = 0.0379. IL-6: 5xFAD vs. WT, *P* = 0.0020; 5xFAD vs. PARP1^−/−^, *P* = 0.0026; 5xFAD vs. 5xFAD/PARP1^−/−^, *P* = 0.0024. C3: 5xFAD vs. WT, *P* = 0.0002; 5xFAD vs. PARP1^−/−^, *P* = 0.0002; 5xFAD vs. 5xFAD/PARP1^−/−^, *P* = 0.0005.

Hippocampal brain sections of WT, 5XFAD, PARP1^−/−^, and 5XFAD/PARP1^−/−^ were immunostained with the microglia marker, ionized calcium-binding adaptor molecule 1 (IBA1), and the astrocyte marker, glial fibrillary acidic protein (GFAP). 5XFAD/PARP1^−/−^ mice exhibited a significant downregulation of astrogliosis (Fig. 5 *A* and *B*) and microgliosis (Fig. 5 *A* and *C*). The mRNA for the neuroinflammatory markers, TNF-α, C1q, IL-1β, IL-6, and C3 mRNA, were significantly upregulated in 5XFAD mice, while they were significantly decreased in 5XFAD/PARP1^−/−^ mice (Fig. 5 *D*–*H*). Thus, our data suggest that loss of PARP1 reduces microglia and astrocyte activation.

### Neurobehavioral Deficits Are Reduced in 5XFAD Mice Lacking PARP1.

**Fig. 6.**
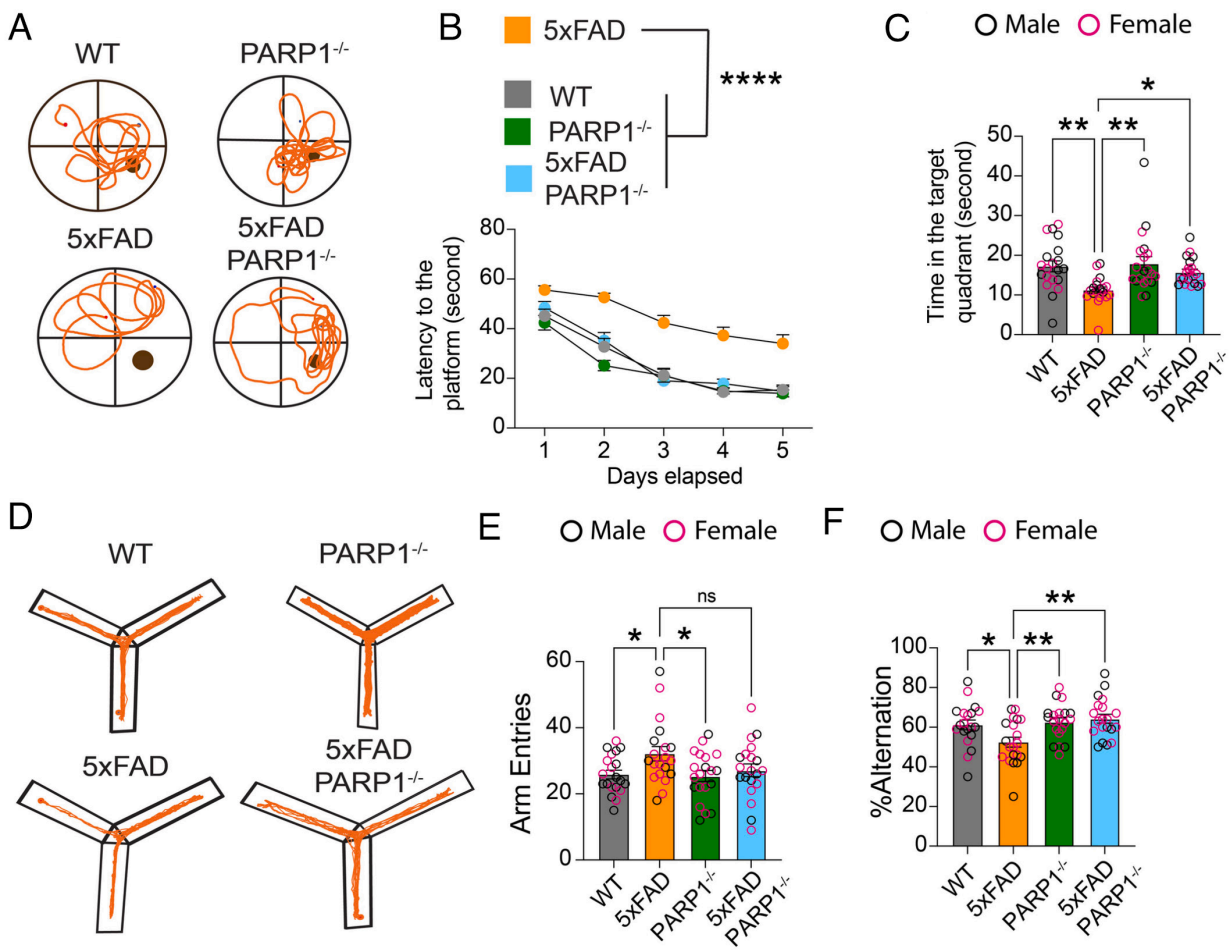
PARP1 deletion improves long term and spatial memory in 5XFAD mice. (*A*–*C*) Morris Water Maze test data. (*A*) Track plot of WT, PARP1^−/−^, 5XFAD, and 5XFAD/PARP1^−/−^ mice on the day of probe trial. (*B*) Latency to find the platform during the training period. Bars represent mean ± SEM (n = 20). Two-way ANOVA was followed by post hoc multiple-comparisons testing comparing 5xFAD to the indicated genotypes: 5xFAD vs. WT, *P* < 0.0001; 5xFAD vs. PARP1^−/−^, *P* < 0.0001; 5xFAD vs. 5xFAD/PARP1^−/−^, *P* < 0.0001. (*C*) Probe trial data: time in the target quadrant. (*D*–*F*) Y-Maze test. (*D*) Trackplot of WT, PARP1^−/−^, 5XFAD and 5XFAD/PARP1^−/−^ mice. (*E*) Number of arm entries. (*F*) Percentage alterations. Bars represent mean ± SEM (n = 20). Two-way ANOVA was followed by post hoc multiple-comparisons testing comparing 5xFAD to the indicated genotypes. Arm entries (*E*): 5xFAD vs. WT, *P* = 0.0373; 5xFAD vs. PARP1^−/−^, *P* = 0.0236; 5xFAD vs. 5xFAD/PARP1^−/−^, *P* = 0.1219 (ns). Percent alternation (*F*): 5xFAD vs. WT, *P* = 0.0323; 5xFAD vs. PARP1^−/−^, *P* = 0.0095; 5xFAD vs. 5xFAD/PARP1^−/−^, *P* = 0.0024.

Neurobehavior of WT, 5XFAD, PARP1^−/−^, and 5XFAD/PARP1^−/−^ was assessed via the Morris water maze, Y-Maze, and open field tests (Fig. 6). Spatial learning and memory were assessed by the Morris water maze task at 9 mo. 5XFAD mice exhibited a significant decline in performance compared to WT and PARP1 KO mice. In contrast, 5XFAD/PARP1^−/−^ showed improved spatial and long-term memory across the 5-d training period (Fig. 6*B*). Probe trial conducted 24 h after the last training trial demonstrated that the 5XFAD/PARP−/− mice spent significantly more time in the target quadrant than the 5XFAD mice (Fig. 6 *A* and *C*), traveled a greater distance in the target quadrant (*SI Appendix*, Fig. S4*A*), and had more entries in the target quadrant (*SI Appendix*, Fig. S4*B*). The Y-maze test was used to assess short-term or working memory. The percentage of alternating behaviors in the 5XFAD mice was significantly lower than that of WT, and PARP1^−/−^ mice (Fig. 6 *D*–*F*). The 5XFAD/PARP1^−/−^ mice significantly prevented the deficits observed in 5XFAD mice. The open field test was performed to evaluate locomotor and anxiety-like behaviors. 5XFAD mice traveled significantly greater distances and spent significantly more time in the periphery than the WT, and PARP1^−/−^ mice (*SI Appendix*, Fig. S4 *C–E*). 5XFAD/PARP1^−/−^ did not exhibit anxiety-like behavior (*SI Appendix*, Fig. S4 *C–E*).

### The Absence of PARP1 Impacts Amyloid Precursor Protein(APP) Levels.

**Fig. 7.**
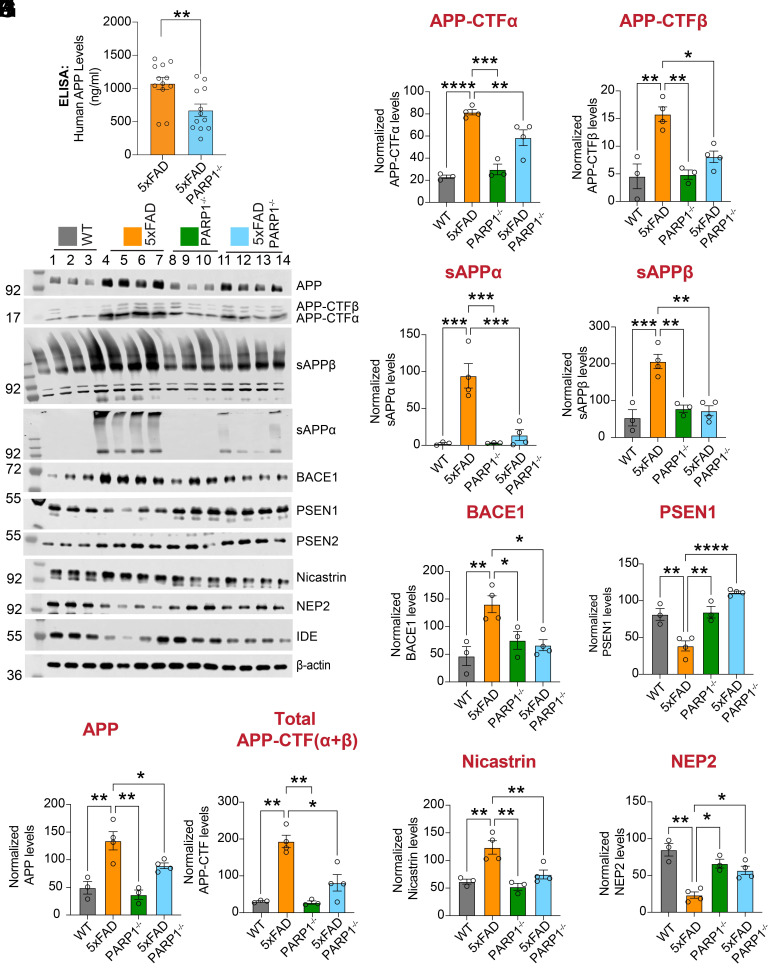
PARP1 deletion impact APP metabolism in 5XFAD mice. (*A*) ELISA quantification of APP levels in cortical lysates from 5XFAD and 5XFAD/PARP1^−/−^ mice. Bars represent mean ± SEM (n = 12). Unpaired two-tailed Student’s *t* test: *P* = 0.0057 (t = 3.063, df = 22). (*B*–*L*) Immunoblotting and quantification of APP and APP-processing intermediates (full-length APP; total APP-CTFs [CTFα + CTFβ]; APP-CTFα; APP-CTFβ; sAPPα; sAPPβ) and APP metabolism–related enzymes (BACE1, PSEN1, PSEN2, nicastrin, and neprilysin [NEP2]) in cortical lysates from WT, PARP1^−/−^, 5xFAD, and 5xFAD/PARP1^−/−^ mice. Bars represent mean ± SEM (n = 3 to 4). Two-way ANOVA was followed by post hoc multiple-comparisons testing comparing 5xFAD to the indicated genotypes. APP: 5xFAD vs. WT, *P* = 0.0043; 5xFAD vs. PARP1^−/−^, *P* = 0.0019; 5xFAD vs. 5xFAD/PARP1^−/−^, *P* = 0.0454. Total APP-CTFs (CTFα + CTFβ): 5xFAD vs. WT, *P* = 0.0004; 5xFAD vs. PARP1^−/−^, *P* = 0.0003; 5xFAD vs. 5xFAD/PARP1^−/−^, *P* = 0.0021. APP-CTFα: 5xFAD vs. WT, *P* < 0.0001; 5xFAD vs. PARP1^−/−^, *P* = 0.0001; 5xFAD vs. 5xFAD/PARP1^−/−^, *P* = 0.0068. APP-CTFβ: 5xFAD vs. WT, *P* = 0.0023; 5xFAD vs. PARP1^−/−^, *P* = 0.0027; 5xFAD vs. 5xFAD/PARP1^−/−^, *P* = 0.0120. sAPPα: 5xFAD vs. WT, *P* = 0.0009; 5xFAD vs. PARP1^−/−^, *P* = 0.0009; 5xFAD vs. 5xFAD/PARP1^−/−^, *P* = 0.0010. sAPPβ: 5xFAD vs. WT, *P* = 0.0008; 5xFAD vs. PARP1^−/−^, *P* = 0.0020; 5xFAD vs. 5xFAD/PARP1^−/−^, *P* = 0.0011. BACE1: 5xFAD vs. WT, *P* = 0.0097; 5xFAD vs. PARP1^−/−^, *P* = 0.0478; 5xFAD vs. 5xFAD/PARP1^−/−^, *P* = 0.0227. PSEN1: 5xFAD vs. WT, *P* = 0.0059; 5xFAD vs. PARP1^−/−^, *P* = 0.0041; 5xFAD vs. 5xFAD/PARP1^−/−^, *P* = 0.0002. Nicastrin: 5xFAD vs. WT, *P* = 0.0026; 5xFAD vs. PARP1^−/−^, *P* = 0.0012; 5xFAD vs. 5xFAD/PARP1^−/−^, *P* = 0.0072. NEP2: 5xFAD vs. WT, *P* = 0.0015; 5xFAD vs. PARP1^−/−^, *P* = 0.0114; 5xFAD vs. 5xFAD/PARP1^−/−^, *P* = 0.0196.

Given that Aβ_1-40_ and Aβ_1-42_ levels were reduced, while APP mRNA levels remained unaltered upon PARP1 depletion in 5XFAD mice (*SI Appendix*, Fig. S2*D*), APP protein expression and processing were subsequently assessed. Human full-length APP ELISA revealed a decrease in APP levels in 5XFAD/PARP1^−/−^ mice (Fig. 7*A*). Immunoblot analyses further showed statistically significant differences in full-length APP and multiple APP processing products, including APP C-terminal fragments (APP-CTFα/β) and the corresponding soluble APP ectodomains (sAPPα/β) (Fig. 7 *B*–*H*). Together, the reduction in full-length APP and the accompanying changes in APP-derived fragments suggest altered APP processing and/or enhanced Aβ clearance, potentially driven by changes in APP-processing and Aβ-degrading proteins.

**Fig. 8.**
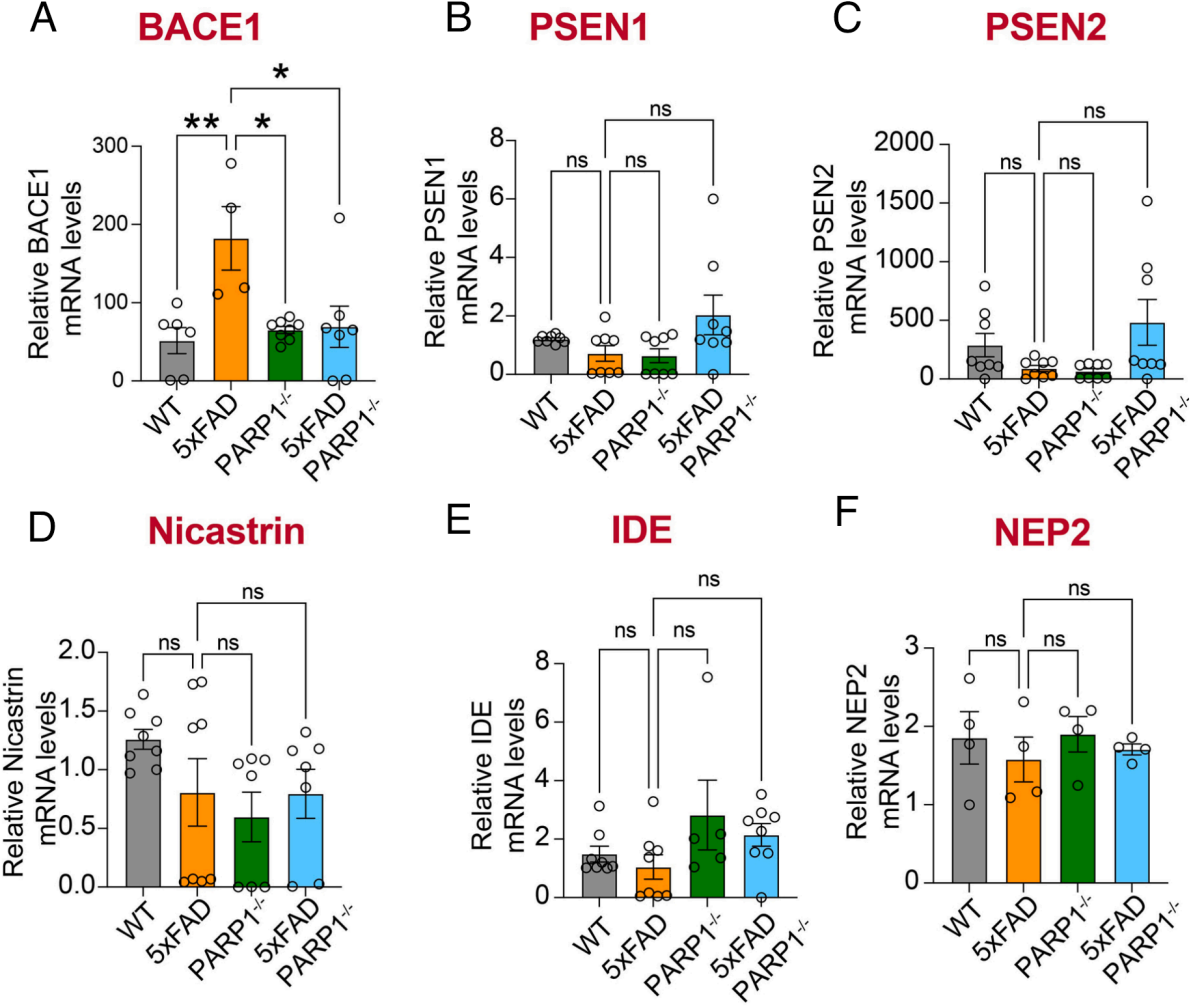
Transcript levels of APP-processing and Aβ-degrading enzymes across WT, PARP1^−/−^, 5xFAD, and 5xFAD/PARP1^−/−^ mice. (*A*–*F*) mRNA levels of BACE1, PSEN1, PSEN2, nicastrin, IDE, and NEP2 in cortical lysates from WT, PARP1^−/−^, 5xFAD, and 5xFAD/PARP1^−/−^ mice, as determined by RT–PCR. Bars represent mean ± SEM (n = 5). Two-way ANOVA was followed by post hoc multiple-comparisons testing comparing 5xFAD to the indicated genotypes. BACE1: 5xFAD vs. WT, *P* = 0.0083; 5xFAD vs. PARP1^−/−^, *P* = 0.0100; 5xFAD vs. 5xFAD/PARP1^−/−^, *P* = 0.0115. PSEN1: 5xFAD vs. WT, *P* = 0.7244 (ns); 5xFAD vs. PARP1−/−, *P* = 0.9982 (ns); 5xFAD vs. 5xFAD/PARP1^−/−^, *P* = 0.0772 (ns). PSEN2: 5xFAD vs. WT, *P* = 0.0872 (ns); 5xFAD vs. PARP1^−/−^, *P* = 0.1247 (ns); 5xFAD vs. 5xFAD/PARP1^−/−^, *P* = 0.3970 (ns). Nicastrin: 5xFAD vs. WT, P = 0.1783 (ns); 5xFAD vs. PARP1^−/−^, *P* = 0.9888 (ns); 5xFAD vs. 5xFAD/PARP1^−/−^, *P* = 0.9982 (ns). IDE: 5xFAD vs. WT, *P* = 0.8537 (ns); 5xFAD vs. PARP1^−/−^, *P* = 0.1167 (ns); 5xFAD vs. 5xFAD/PARP1^−/−^, *P* = 0.2762 (ns). NEP2: 5xFAD vs. WT, *P* = 0.7908 (ns); 5xFAD vs. PARP1^−/−^, *P* = 0.7115 (ns); 5xFAD vs. 5xFAD/PARP1^−/−^, *P* = 0.9693 (ns).

5XFAD/PARP1^−/−^ mice showed a significant decrease in BACE1 protein levels (Fig. 7 *B* and *I*) and BACE1 mRNA levels (Fig. 8*A*). Among the components of γ-secretase complex, PSEN1 protein levels were upregulated (Fig. 7 *B* and *J*) and nicastrin protein levels were reduced (Fig. 7 *B* and *K*), while PSEN2 protein levels remained unchanged (Fig. 7*B* and *SI Appendix*, Fig. S5*A*). At the mRNA level PSEN1, PSEN2 and nicastrin showed no significant differences (Fig. 8 *B*–*D*). Enzymes involved in Aβ degradation, such as Neprilysin (NEP2), were upregulated in 5XFAD/PARPP1^−/−^ mice, while the insulin-degrading enzyme (IDE), remained unchanged (Fig. 7 *B* and *L* and *SI Appendix*, Fig. S5*B*). No statistical changes in mRNA levels were observed for IDE and NEP2 (Fig. 8 *E* and *F*). In summary, these results suggest a reduced APP processing rate combined with enhanced Aβ degradation as a potential mechanism driving the decreased Aβ plaque load in 5XFAD/PARP1^−/−^ mice.

## Discussion

The major findings of this study demonstrate that PAR levels are significantly elevated in the CSF of patients with MCI and AD, with PAR levels negatively correlating with the Aβ_42/40_ ratio, a biomarker with strong concordance to amyloid PET positivity (24, 25). Mechanistic investigations revealed that oligomeric Aβ_1-42_, but not Aβ_1-40_, activates PARP1 in primary cortical neurons, resulting in increased PAR synthesis, DNA damage, and neuronal death—effects that are prevented by both pharmacological inhibition and genetic ablation of PARP1. In vivo, genetic deletion of PARP1 in the 5XFAD mouse model of AD led to a reduction in amyloid plaque burden, preservation of synaptic density, prevention of neuronal loss, and significant attenuation of glial activation and neuroinflammation. Importantly, PARP1 deficiency also rescued learning and memory deficits in these mice. At the molecular level, PARP1 ablation resulted in reduced BACE1 expression, altered γ-secretase complex composition, and upregulation of the Aβ-degrading enzyme NEP2, collectively shifting APP metabolism toward decreased Aβ production and enhanced clearance (26, 27). These integrated results identify PARP1 as an important modifier of amyloid-driven neurotoxicity, neuroinflammation, and cognitive decline in AD, and highlight its potential as a disease-modifying therapeutic target.

PARP1 is a ubiquitously expressed nuclear enzyme with established roles in DNA damage responses and in regulating inflammatory transcriptional programs in multiple cell types, including glia. In this study, we focused on neurons to test whether PARP1 is a critical mediator of Aβ-driven neurotoxicity and to determine whether PARP1 loss preserves neuronal integrity in vivo, using the 5XFAD model because it exhibits early, quantifiable neuron loss and neurodegenerative changes. At the same time, prior work in hAPP-J20 mice and in vitro has shown that PARP1 also modulates microglial responses to Aβ (28), and we similarly observe reduced astrocytosis, microgliosis, and inflammatory gene expression in 5XFAD/PARP1^−/−^ mice. These convergent findings support a model in which neuronal and glial PARP1 signaling both contribute to amyloid-driven pathology, and future studies using cell type–specific manipulation of PARP1 will be important to define their relative and potentially interacting roles in AD.

We report PARP1 loss alters APP metabolism by reducing BACE1 (β-secretase) expression and modifying γ-secretase complex composition, thereby decreasing Aβ production. In addition, PARP1 knockout enhances Aβ clearance through upregulation of NEP2, an Aβ-degrading enzyme, without affecting IDE. These findings suggest that PARP1 orchestrates both amyloidogenic processing and clearance pathways, and its loss shifts the balance toward reduced amyloid accumulation. The observed changes in BACE1 and γ-secretase subunits are consistent with PARP1’s known roles in regulating stress and inflammatory signaling, particularly via NF-κB, which can drive BACE1 transcription in response to Aβ-induced oxidative stress (29, 30).

PARP1 deficiency also led to coordinated changes in the γ-secretase complex and Aβ clearance pathways that together may help explain the reduction of the amyloid burden observed in 5XFAD/PARP1^−/−^ mice. Specifically, we observed an increase in PSEN1 protein, the primary catalytic subunit of γ-secretase, alongside a significant downregulation of nicastrin at both the protein and mRNA level, while PSEN2 protein remained unchanged. Since PSEN1 is essential for most Aβ generation (31), its upregulation may reflect a compensatory response to maintain γ-secretase activity; however, the concomitant loss of nicastrin—critical for substrate recognition—would be expected to impair complex assembly and function, ultimately resulting in reduced Aβ production despite increased PSEN1. This is further complemented by the observed decrease in BACE1, collectively shifting APP processing toward a less amyloidogenic route. In parallel, the absence of PARP1 enhanced Aβ clearance, as evidenced by increased levels of the Aβ-degrading enzyme, NEP2, without corresponding changes in its mRNA, suggesting post transcriptional regulation or protein stabilization (32). Given that NEP2 activity is known to mitigate amyloid pathology, its upregulation in PARP1-deficient animals would further accelerate Aβ catabolism. These integrated alterations—diminished amyloidogenic processing and enhanced degradation suggest how PARP1 ablation may orchestrate a multifaceted reduction in amyloid burden, extending prior evidence that chronic PARP1 activation exacerbates amyloid pathology and cognitive decline, while its inhibition confers protection in Alzheimer’s models.

Collectively, these results suggest that PARP1 may play an important role in mediating Aβ-driven neurotoxicity, amyloid pathology, neuroinflammation, and cognitive impairment in AD models. The integration of human biomarker data, mechanistic cellular studies, and in vivo genetic evidence supports further investigation of PARP1 as a potential target for disease-modifying therapeutic strategies in AD.

## Materials and Methods

### Animal.

5XTg FAD (MMRRC Strain #034840-JAX) mice were obtained from the Jackson Laboratories (Bar Harbor, ME). The lab-maintained PARP1^−/−^ mice line was used to cross 5XTg FAD and PARP1^−/−^ together. After two generations, the desired littermates were aged and used for further experimentation. NIH Guide for the Care and Use of Experimental Animals and Johns Hopkins University Animal Care and Use Committee were followed for all housing, breeding, and subsequent procedures.

### Antibodies.

Primary antibodies used for Western blotting and immunochemistry are listed below. These included antibodies against APP, APP-CTF, sAPPβ, Nicastrin, BACE1, PSEN1, PSEN2, NEP2, IDE, β-Actin, IBA1, GFAP, Aβ (6E10 and 4G8), NeuN, RTN3, PSD95, PARP1, PAR, and γH2AX. All antibodies were obtained from commercial sources as detailed, except for the PAR antibody, which was prepared in-house. All commercial antibodies are listed in *SI Appendix*, Table S1.

### Human CSF Samples and PAR ELISA.

Participants in the Johns Hopkins University BIOCARD study (33) and at the Cleveland Clinic underwent annual evaluations, including detailed medical history, physical examination, and neuropsychological testing. All human studies were approved by the Johns Hopkins Medicine IRB protocol number NA_00027232 and the Cleveland Clinic IRB approval number 14-604. All subjects provided informed consent. CSF was collected and CSF specimens were processed within 1 h of collection: samples were centrifuged, divided into aliquots, and stored at –80 °C at either the Cleveland Clinic Lou Ruvo Center for Brain Health Biobank or the Johns Hopkins University repository. For PAR quantification, two monoclonal anti-PAR antibodies (clones #19 and #25) were employed in an ELISA format. Ninety-six–well plates (NUNC, Cat. #46051) were coated overnight at 4 °C with clone #19 (5 µg/mL) as the capture antibody. Purified PAR standards (0 to 200 nM) and CSF samples from control or PD subjects were added and incubated for 1 h at room temperature. Wells were washed five times with PBST (0.05% Tween-20 in PBS), followed by a 1-h incubation with biotinylated clone #25 as the detection antibody. Signal development was achieved using HRP-conjugated streptavidin (Thermo Scientific), yielding a lower detection limit of ~3 pM and saturation at 50 nM.

### Primary Cortical Neuron Culture and Treatment.

Primary cortical neurons were isolated from embryonic day-16 WT or PARP1^−/−^ mouse embryos as previously described (5). Cells were seeded in Neurobasal medium supplemented with B-27, 0.5 mM L-glutamine, and 100 U/mL penicillin–streptomycin (Invitrogen, Carlsbad, CA). At DIV 7, cultures were pretreated for 1 h with one of the following: ABT-888 (veliparib) at 1 µM, AG-014699 (rucaparib) at 1 µM, BMN 673 (talazoparib) at 1 µM. Thereafter, 1 µM oAβ_1-42_ or 1 µM oAβ_1-40_ were added, and cells were incubated for the indicated durations before analysis by cell-death assays or biochemical methods.

### Synthetic Oligomeric oAβ_1-42_ Preparation.

Synthetic Aβ_1-42_ oligomers were generated from lyophilized monomers (rPeptide, Bogart, GA). Briefly, HFIP-treated Aβ_1-42_ was first dissolved in DMSO, then diluted into PBS to the desired concentration. The solution was incubated at 4 °C for 24 h to allow oligomer formation and subsequently stored at –80 °C. Prior to use, samples were centrifuged at 12,000 × g for 10 min, and the cleared supernatant was collected as the oligomeric Aβ (ADDLs). Oligomerization was confirmed by Western blot analysis (34).

### Cell Death and Viability Assessment.

Primary cortical neurons were exposed to 1 μM oAβ_1–42_ (oAβ1–42) for 48 h. PARP inhibitors, including Talazoparib (BMN 673; LT-673, Catalog No. S7048), AG-14361 (Catalog No. S2178), and Veliparib (ABT-888; NSC 737664, Catalog No. S100), were added 30 min prior to oAβ_1–42_ treatment. Cell death was quantified by costaining with 7 μM Hoechst 33342 and 2 μM propidium iodide (PI) (Invitrogen), followed by automated image acquisition and analysis on a Zeiss microscope using Axiovision 4.6 software (Carl Zeiss, Dublin, CA). Subsequently, Alamar Blue reagent (Invitrogen) was added, and cell viability was measured fluorometrically (λ^ex^ = 570 nm, λ^em^ = 585 nm) as described previously (35).

### Tissue Extraction and Immunoblot Analysis.

Frozen cortical tissue from dissected brains were homogenized in Tris-buffered saline (TBS) homogenization buffer (20 μL/mg). The sample was centrifuged at 100,000 × g for 1 h at 4 °C using Optima TLX Ultracentrifuge (Beckman Coulter). The supernatant was transferred to a prechilled Eppendorf and pellet was resuspended in TBS buffer containing 1% Triton X-100 (20 μL/mg). This time the sample was sonicated and then centrifuged at 100,000 × g for 1 h at 4 °C using Optima TLX Ultracentrifuge. The supernatant was saved as TBSX soluble. The remaining pellet was finally extracted using 70% formic acid. Following similar rounds of sonication and centrifugation, supernatant was saved as 70% formic acid soluble. This protocol is adapted from Youmans et al. (36). The 70% formic acid extracts were further neutralized by adding 20 volumes of 1 M Tris, pH 11 (37). For immunoblotting, proteins were resolved by SDS–PAGE using Tris–glycine gels for all targets, except for APP-CTF experiments, which were run on Tris–Tricine gels to improve separation of low-molecular-weight fragments.

### Aggregated Aβ Extraction.

Mouse cortical tissue was lysed using an ice-cold buffer consisting of 10 mM Tris-HCl (pH 7.5), 150 mM NaCl, 5 mM MgCl2, 0.5 mM DTT, 100 µg/mL cycloheximide, along with protease and RNase inhibitors, and 0.05% sodium deoxycholate. The homogenization was performed with a Dounce homogenizer. The resulting homogenates were incubated at 4 °C for 20 min and then cleared through centrifugation at 10,000 rpm for 10 min at 4 °C. The supernatants were collected, quantified using a BCA assay, and normalized for total protein content. A sucrose cushion was created by dissolving 2 g of sucrose in 4.7 mL of lysis buffer. For each sample, 900 µL of this cushion was layered beneath 600 µL of the normalized lysate, followed by centrifugation at 70,000 rpm for 2 h at 4 °C. After ultracentrifugation, the supernatants were carefully removed, and the pellets were resuspended in 60 µL of lysis buffer. The enrichment of aggregates was verified through immunoblotting before proceeding to mass spectrometry.

### Amyloid-β ELISA.

ELISAs to quantify distinct amyloid species, human Aβ^1–^^42^ (Thermo Fisher Scientific, Cat. #KHB3441), human Aβ^1–^^40^ (Thermo Fisher Scientific, Cat. #KHB3481), and human APP (Thermo Fisher Scientific, Cat. #KHB0051), were performed according to the manufacturer’s instructions. Cortical tissue was used for all immunoblot and ELISA analyses. Details of tissue lysate preparation are described in the Tissue Extraction and Immunoblot Analysis section of *Materials and Methods*. For APP ELISA assays, TBSX-soluble fractions were used.

### Immunohistochemistry and Immunofluorescence.

All mice were perfused with PBS and dissected to preserve half the hemisphere of the brain for immunostaining and other half for western blotting. The half saved for immunostaining was fixed overnight with 4% PFA followed by transfer to 30% sucrose for cryoprotection, where the brains remained until sectioned. Sample brains were sectioned at 50 μm thickness. Brains sections were then processed for immunostaining. By first incubating the sample in antigen retrieval buffer (Thermo catalog-00-4956-58). This was followed by three PBS wash steps. The sections were then permeabilized using 0.3× triton X-100 contained in 10% goat serum. After this, the sections were blocked for an hour. Primary antibody incubation was performed overnight in a cold room. The next day, sections were PBS washed three times before incubating with secondary antibody for an hour at room temperature. After three washes, the samples were mounted using a mounting media containing DAPI. Similar steps were followed for immunofluorescence of primary neurons. For the Thioflavin S staining procedure, each brain section was treated with a 500 µM solution of Thioflavin S (ThS, Sigma-Aldrich, USA) in 50% ethanol for a duration of 7 min followed by ethanol washes and were subsequently mounted using mounting media. For Nissl staining, sections were counterstained with Nissl (0.09% thionin). The cell counting was performed using stereo investigator software. Immunofluorescence imaging was performed using confocal microscope- LSM880. Signal intensity and plaque counting was performed using ImageJ software (5).

### Real-Time qPCR (RT-qPCR).

For the RT-qPCR procedure, total RNA was isolated utilizing TRIzol, following the manufacturer’s protocol. One microgram of the extracted RNA underwent reverse transcription with the High-Capacity cDNA Reverse Transcription Kit. Gene expression analysis was performed using SYBR Green-based RT-qPCR on an ABI ViiA 7 system (Applied Biosystems, Foster City, CA), with results normalized to GAPDH levels. Forward (F) and reverse (R) primers were (5′ to 3′, human sequences unless stated otherwise) are listed in *SI Appendix*, Table S2.

### Behavioral Tests.

#### Morris water maze.

The Morris water maze was performed as published by Park et al. (38) with minor modifications. A circular pool (120 cm in diameter and 35 cm in height) was filled with water containing a nontoxic water-soluble white dye. The pool was split into four equal quadrants. A platform (8 cm in diameter) was randomly placed in one of the quadrants such that it does not appear visible (1 cm below the water surface), possibly in the center of that quadrant. Visual cues were attached around the quadrants to act as spatial references. A day before the trials mice were given a 60 s swimming training in the absence of a platform. This is followed by a 5-d training period wherein the mice swam three time (trials) a day, with an intertrial interval of 30 min, to develop memories to allocate the hidden platform, termed as escape latency. During this period, mice were given 60 s to find the platform, if successful within this time frame the test automatically ends, but if they are unsuccessful, they are made to stand on the platform for 10 s. On the final day- probe trial, the platform was removed. The mice were again given 60 s to swim. The time and distance spent in the target quadrant (previously containing the platform) was recorded. For the entirety of this experiment, ANY-maze software (ANY-maze system, Wood Dale, IL) was used for recording.

#### Y-maze spontaneous alteration.

The Y-maze spontaneous alteration was performed as published by Park et al. (38) with minor modifications. Mice were given 5 min to freely explore a Y-maze (40 × 8 × 15 cm). Using ANY-maze software the number of arm entries and percent alternations by the mice were recorded. An entry was considered legitimate only when all four limbs of the mice were within the arm.

#### Open field test.

The Open field test was performed as described (39). Using Photobeam Activity System/PAS software (SD instruments), a rectangular box (40 cm × 40 cm × 40 cm) was digitally subdivided into 36 (6 × 6) identical sectors (6.6 cm × 6.6 cm), which was further subdivided into peripheral and central sectors. The mouse was placed inside this box in the dark and its movement was monitored via software for 30 min (5 min each six cycles). Between mouse change, the apparatus was thoroughly cleaned using Vinoba. The time spent in periphery vs. the center was collected and graphed as a marker for anxiety.

### Statistical Analysis and Quantification.

Quantifications on immunoblots and immunofluorescent data was performed using ImageJ. For PSD95 density analysis, a Matlab script published in a previous paper was repurposed for analysis (40). Each color channel was first converted to binary signal. The Aβ plaque image was used to outline the plaque using binary boundary detection. The code then dilated this boundary by 30 µm and applied the outlines onto the thresholded red channel to compute the area of the PS95 signal as a percentage of total area in each annulus.

All data are represented as mean ± SEM. At least 3 independent experiments were performed for in vitro and immunofluorescence experiments. For behavioral experiments, the sample size (n) was about 20 mice. Statistical analysis was performed using GraphPad Prism 9 and CSF associated correlation analysis was performed using IBM SPSS. Differences between 2 means were calculated using an unpaired two-tailed Student *t* test, and among multiple means using ANOVA followed by Tukey’s post hoc test. All *P*-values from the datasets were consolidated into a single table (Dataset S2) and *P*-values are also reported directly with the corresponding figure legends for clarity.

## Supplementary Material

Appendix 01 (PDF)

Dataset S01 (XLSX)

Dataset S02 (XLSX)

## Data Availability

Original data and data source files data have been deposited in DRYAD (DOI: 10.5061/dryad.1rn8pk174) (41).
