# Supplementary material for: PARP1 deficiency mitigates amyloid pathology, neurodegeneration, and cognitive decline in a familial Alzheimer’s disease model
Source: Proc Natl Acad Sci U S A. 2026 May 15;123(20):e2525028123. doi: 10.1073/pnas.2525028123 (PMC13187709; doi:10.1073/pnas.2525028123)
Supplement: Supplementary file 1 — Appendix 01 (PDF) [file pnas.2525028123.sapp.pdf]

PNAS

Supporting Information for

PARP1 Deficiency Mitigates Amyloid Pathology, Neurodegeneration, and Cognitive Decline in a Familial Alzheimer's Disease Model

Aanishaa Jhaldiyal, Manisha Kumari, Lauren C. Guttman, Trupti Tripathi, Mohammed Repon Khan, Justin Wang, Devanik Biswas, Abhishek Pasupuleti, Akansha Aggarwal, Shraddha Pandya, Shih-Ching Chou, Nikhil Panicker, Abhay Monghekar, Marilyn Albert, Lynn M. Bekris, James B. Leverenz, Tae-In Kam, Ted M. Dawson and Valina L. Dawson

Corresponding authors: Ted M. Dawson, Email: tdawson@jhmi.edu or Valina L. Dawson, Email: vdawson@jhmi.edu

This PDF file includes:

Figures S1 to S5
Table S1 to S2
SI Reference

Other supporting materials for this manuscript include the following:

Datasets S1 to S2

Figures

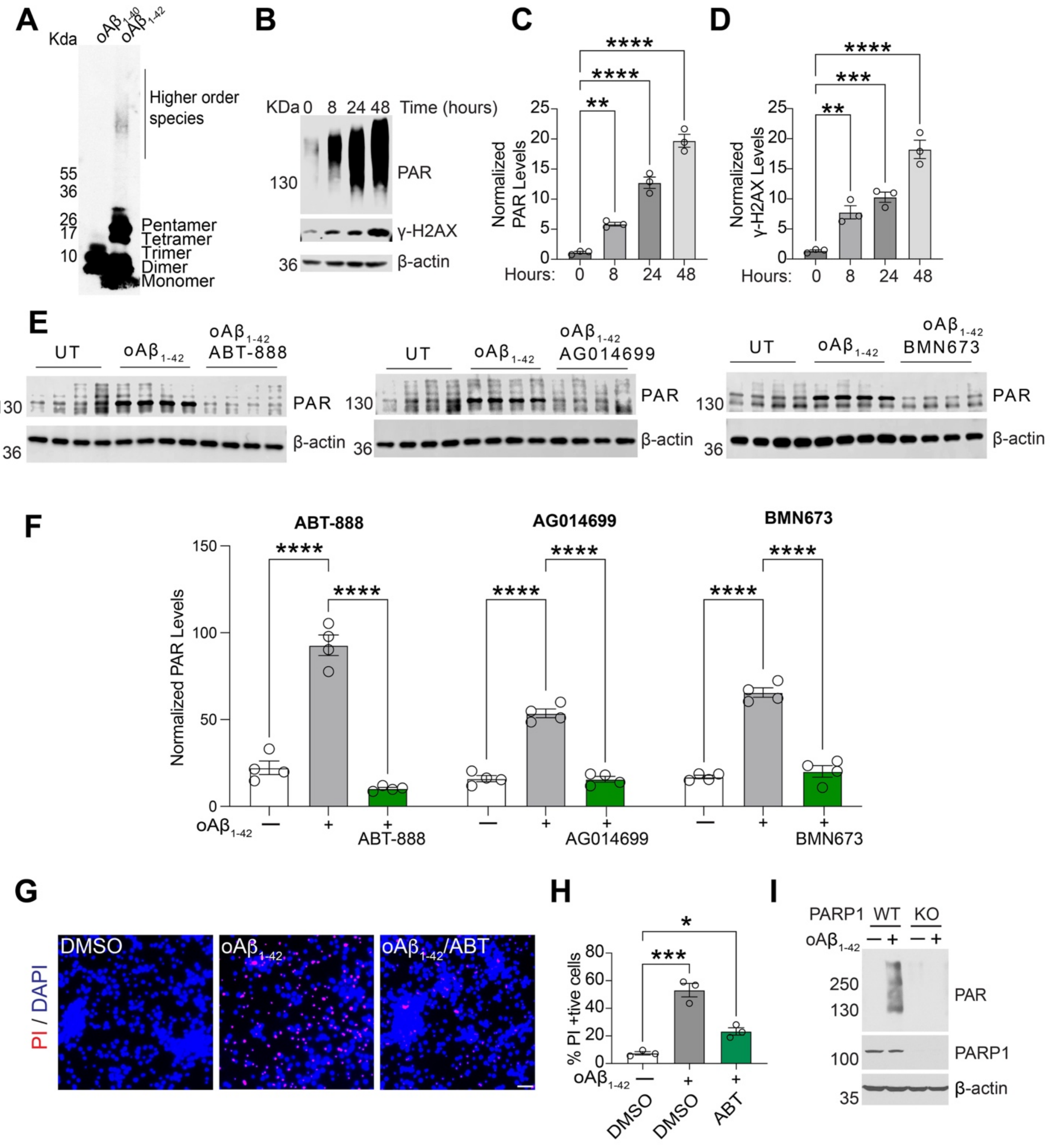

Figure S1: $oA\beta_{1-42}$ causes time dependent increase in PAR levels.
A) $A\beta_{1-40}$ and $A\beta_{1-42}$ oligomers visualized using immunoblot.
B-D) PAR and γ-H2AX levels increase in a time-dependent manner in primary neurons treated with 1 μM $oA\beta_{1-42}$. (B) Representative immunoblot of PAR and γ-H2AX at the indicated time points. (C) Quantification of PAR levels over time. (D) Quantification of γ-H2AX levels over time. Bars represent mean ± SEM (n = 3). One-way ANOVA with Tukey's post hoc test. Bars represent mean ± SEM (n = 3). One-way ANOVA was followed by post hoc multiple-comparisons testing

versus the 0 h time point. PAR (C): 0 vs 8 h, $P = 0.0050$; 0 vs 24 h, $P < 0.0001$; 0 vs 48 h, $P < 0.0001$. γ-H2AX (D): 0 vs 8 h, $P = 0.0060$; 0 vs 24 h, $P = 0.0008$; 0 vs 48 h, $P < 0.0001$.
E,F) Pharmacological inhibition of PARP reduces Aβ-induced PARP activity in primary cortical neurons. Primary cortical neurons were treated with 1 μM oAβ1–42 for 24 h with or without pretreatment with PARP inhibitors (ABT-888, BMN 673, or AG-014699). (E) Representative immunoblot of poly(ADP-ribose) (PAR). (F) Quantification of PAR levels. Pretreatment with ABT-888, AG-014699, or BMN 673 reduced oAβ1–42–induced PAR accumulation, consistent with inhibition of PARP enzymatic activity. Bars represent mean ± SEM (n = 3). One-way ANOVA was followed by post hoc multiple-comparisons testing: ABT-888: oAβ1–42 vs UT, $P < 0.0001$; oAβ1–42 vs oAβ1–42 + ABT-888, $P < 0.0001$. AG-014699: oAβ1–42 vs UT, $P < 0.0001$; oAβ1–42 vs oAβ1–42 + AG-014699, $P < 0.0001$. BMN 673: oAβ1–42 vs UT, $P < 0.0001$; oAβ1–42 vs oAβ1–42 + BMN 673, $P < 0.0001$.
G,H) 1 μM ABT888 protects against oA$\beta_{1\text{-}42}$ induced neurotoxicity. (D) Representative images of DAPI and propidium iodide (PI) staining from primary cortical neurons pre-treated with ABT-888 for 1 h, followed by further incubation with oA$b_{1\text{-}42}$ for 2 days. (E) Quantification of cell death. Bars represent mean ± SEM. Two-way ANOVA was followed by post hoc multiple-comparisons testing: DMSO vs oAβ1–42, $P = 0.0001$; DMSO vs oAβ1–42 + ABT-888, $P = 0.0247$.
I) Inhibition of PAR accumulation in primary cortical neurons of PARP1$^{-/-}$ versus WT neuronal culture.

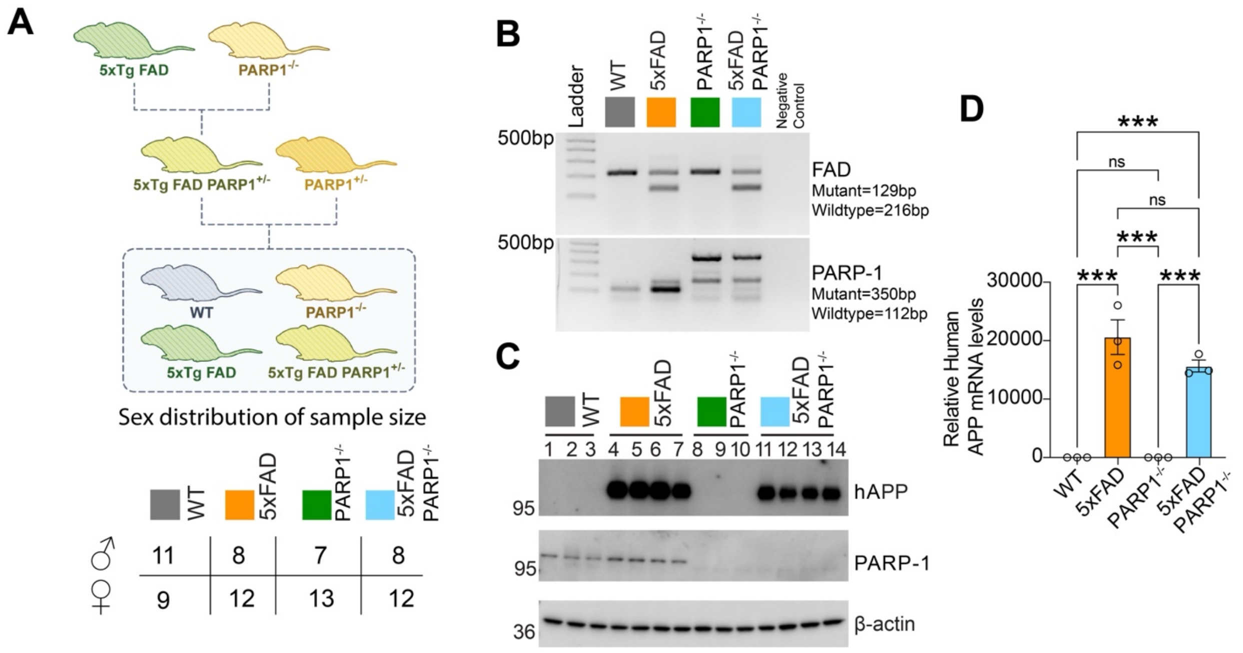

Figure S2: Generation of 5XFAD/PARP1$^{-/-}$ mice.
A-C) (A) Breeding scheme of PARP1$^{-/-}$ with 5XFAD mice is shown, along with the numbers of male and female mice used for further behavioral analysis within each genotypes. Data is shown along with (B) representative genotyping data verification and (C) immunoblot data. Genotyping was performed using primer sequence provided by Jackson Labs.
D) Human APP mRNA levels do not differ significantly between 5xFAD and 5xFAD/PARP1$^{-/-}$ mice. RT–PCR was performed to quantify human APP mRNA in WT, PARP1$^{-/-}$, 5xFAD, and 5xFAD/PARP1$^{-/-}$ mice. Bars represent mean ± SEM (n = 3). Two-way ANOVA was followed by Tukey's multiple comparisons test: WT vs 5xFAD, P = 0.0002; WT vs PARP1$^{-/-}$, P > 0.9999 (ns); WT vs 5xFAD/PARP1$^{-/-}$, P = 0.0009; 5xFAD vs PARP1$^{-/-}$, P = 0.0002; 5xFAD vs 5xFAD/PARP1$^{-/-}$, P = 0.1606 (ns); PARP1$^{-/-}$ vs 5xFAD/PARP1$^{-/-}$, P = 0.0009.

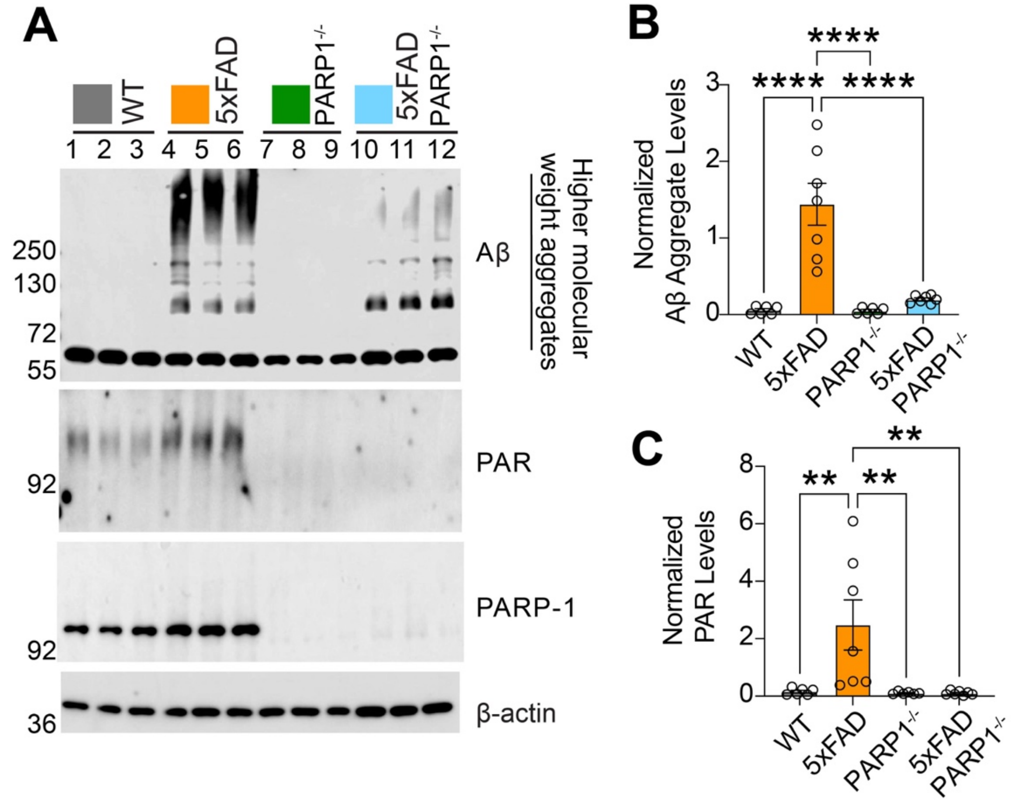

Figure S3: PARP1 deletion mitigates aggregated Aβ and PAR accumulation in 5xFAD mice. (A) Immunoblot and quantification of extracted aggregated Aβ and PAR in WT, PARP1−/−, 5xFAD, and 5xFAD/PARP1−/− mice. Representative immunoblots show aggregated Aβ and poly(ADP-ribose) (PAR) levels, with corresponding quantification of (B) aggregated Aβ and (C) PAR. Bars represent mean ± SEM (n = 6-7). Two-way ANOVA was followed by post hoc multiple-comparisons testing comparing 5xFAD to the indicated genotypes: Aggregated Aβ (B): 5xFAD vs WT, $P < 0.0001$; 5xFAD vs PARP1−/−, $P < 0.0001$; 5xFAD vs 5xFAD/PARP1−/−, $P < 0.0001$. PAR (C): 5xFAD vs WT, $P = 0.0092$; 5xFAD vs PARP1−/−, $P = 0.0079$; 5xFAD vs 5xFAD/PARP1−/−, $P = 0.0064$.

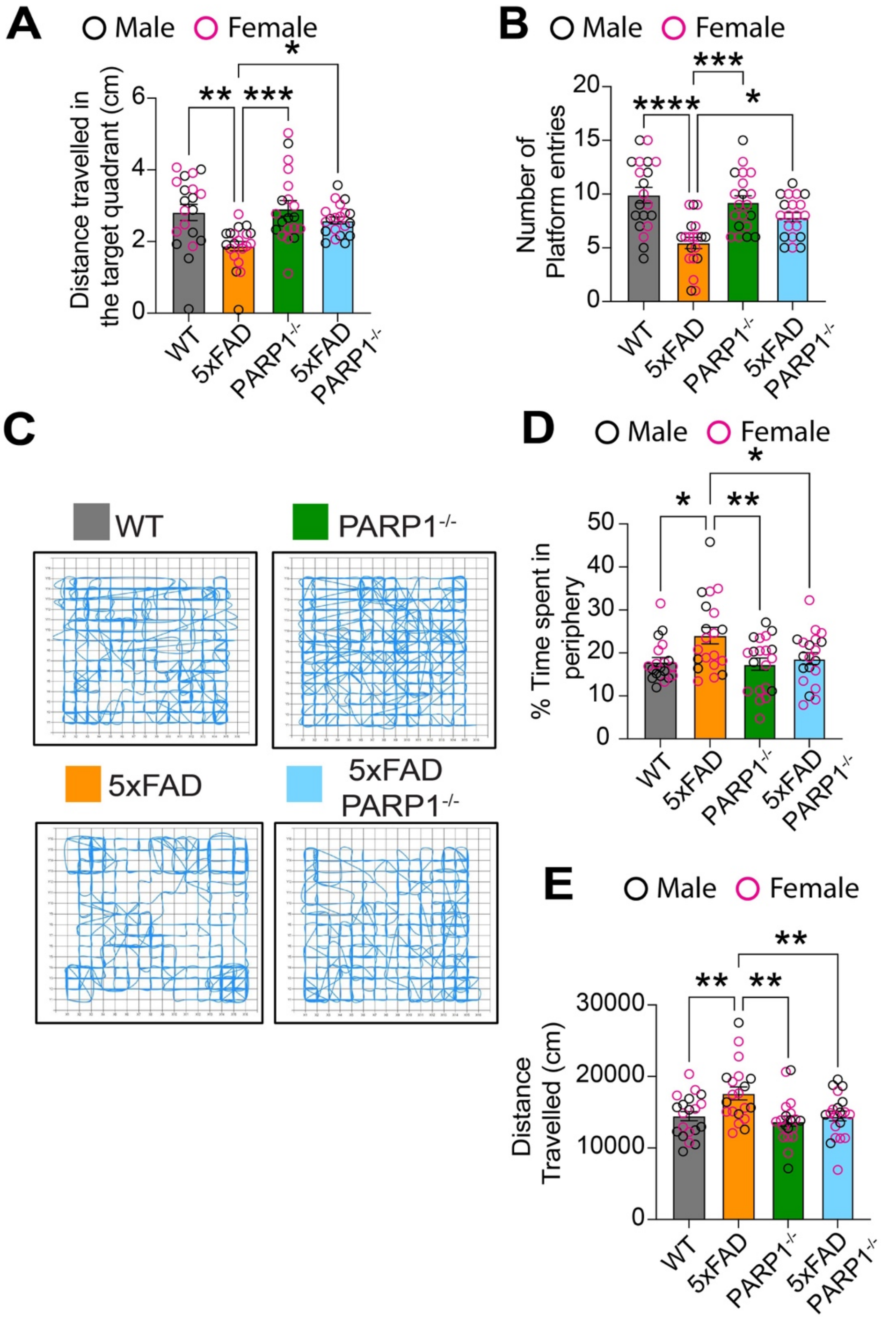

Figure S4: PARP1$^{-/-}$ reduces anxiety like behavior in 5XFAD mice.
A,B) Additional MWM Probe trial data: A) distance in the target quadrant and B) number of platform entries. Bars represent mean ± SEM (n = 20). Two-way ANOVA was followed by post hoc multiple-comparisons testing comparing 5xFAD to the indicated genotypes. Distance in target quadrant (A): 5xFAD vs WT, $P = 0.0021$; 5xFAD vs PARP1−/−, $P = 0.0006$; 5xFAD vs 5xFAD/PARP1−/−, $P = 0.0229$. Platform entries (B): 5xFAD vs WT, $P < 0.0001$; 5xFAD vs PARP1−/−, $P = 0.0001$; 5xFAD vs 5xFAD/PARP1−/−, $P = 0.0197$.

C-E) Open field test. C) Trackplot of WT, PARP1$^{-/-}$, 5XFAD and 5XFAD/PARP1$^{-/-}$ mice. D) Percentage of time spent in the periphery E) Distance travelled by the mice in open field test. Bars represent mean ± SEM (n = 20). Two-way ANOVA was followed by post hoc multiple-comparisons testing comparing 5xFAD to the indicated genotypes. Time in periphery (D): 5xFAD vs WT, P = 0.0022; 5xFAD vs PARP1−/−, P = 0.0010; 5xFAD vs 5xFAD/PARP1−/−, P = 0.0020. Distance traveled (E): 5xFAD vs WT, P = 0.0085; 5xFAD vs PARP1−/−, P = 0.0011; 5xFAD vs 5xFAD/PARP1−/−, P = 0.0090.

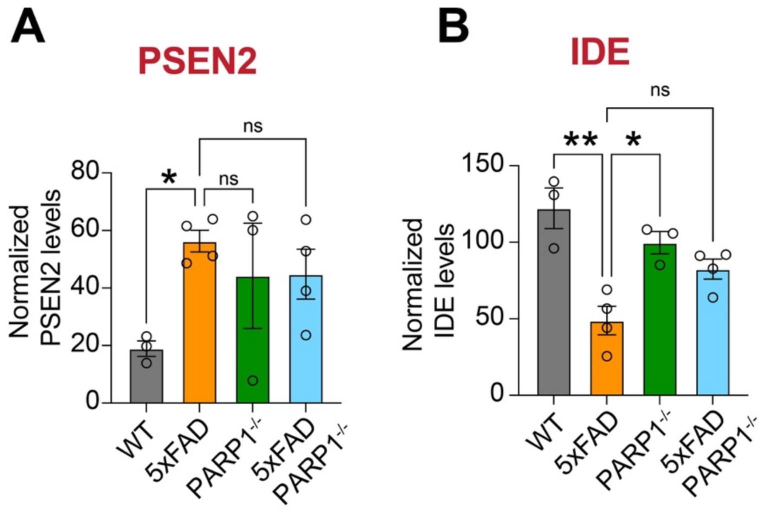

Figure S5: PSEN2 and IDE protein levels are not significantly altered between 5XFAD and 5XFAD/PARP1$^{-/-}$ mice.
A, B) Quantification of PSEN2 (A) and IDE (B) from the representative immunoblot in Fig. 7B of cortical lysates from WT, PARP1−/−, 5xFAD, and 5xFAD/PARP1−/− mice. Bars represent mean ± SEM (n=3-4). Two-way ANOVA was followed by post hoc multiple-comparisons testing comparing 5xFAD to the indicated genotypes. PSEN2: 5xFAD vs WT, P = 0.0872 (ns); 5xFAD vs PARP1−/−, P = 0.1247 (ns); 5xFAD vs 5xFAD/PARP1−/−, P = 0.3970 (ns). IDE: 5xFAD vs WT, P = 0.8537 (ns); 5xFAD vs PARP1−/−, P = 0.1167 (ns); 5xFAD vs 5xFAD/PARP1−/−, P = 0.2762 (ns).

Tables

Table S1. Antibodies

Antibody	Catalog Number	Company / Source
APP	14-9749-82	Thermo Fisher Scientific
APP-CTF	A8717-25UL	Sigma-Aldrich
Anti-Amyloid Precursor Protein antibody [Y188]	ab32136	Abcam
sAPPβ	18957	IBL America
Nicastrin (Rabbit mAb)	9447	Cell Signaling Technology
BACE1	PA1-757	Thermo Fisher Scientific
PSEN1 (D39D1, Rabbit mAb)	5643	Cell Signaling Technology
PSEN2 (D30G3, Rabbit mAb)	9979	Cell Signaling Technology
NEP2	AB5458	Millipore
IDE	921203	BioLegend
β-Actin–Peroxidase (Mouse mAb)	A3854	Sigma-Aldrich
IBA1	ab5076	Abcam
GFAP	ab7260	Abcam
Aβ (6E10)	803004	BioLegend
Aβ (4G8)	800712	BioLegend
NeuN (clone 13E6, Rabbit mAb, ZooMAb®)	ZRB377	Sigma-Aldrich
RTN3 (Polyclonal)	12055-2-AP	Proteintech
PSD95	ab18258	Abcam
PARP1 (Clone 4C10-5, Mouse mAb)	556494	BD Pharmingen™, BD Biosciences
PAR (poly-ADP-ribose)	In-house preparation	—
γH2AX (phospho-S139, clone 9F3)	ab26350	Abcam

Antibodies used in the study including source and Catalog number.

Table S2. Forward and reverse primer sequences

	Primer Name	Sequence	Source
1	BACE_F	GGAACCCATCTCGGCATCC	(1)
2	BACE_R	TCCGATTCCTCGTCGGTCTC	(1)
3	PSEN1_F	ATACCTGCACCTTTGTCCTACT	(1)
4	PSEN1_R	GCTCAGGGTTGTCAAGTCTCT	(1)
5	PSEN2_F	GAAGACTCCTACGACAGTTTTGG	(1)
6	PSEN2_R	CACCAGGACGCTGTAGAAGAT	(1)
7	Ncstn_F	TCCGTGGTACTGGCAGGATT	(1)
8	Ncstn_R	CCCCTGTATCCCCACTAATTGA	(1)
9	IDE_F	AATCCGGCCATCCAGAGAATA	(1)
10	IDE_R	GGGTCTGACAGTGAACCTATGT	(1)
15	NEP_F	TCCTGACTATCATAGCGGTGAC	(2)
16	NEP_R	GACGTTGCGTTTCAACCAGC	(2)
17	APP_F	CAAGCAGTGCAAGACCCATC	(1)
18	APP_R	AGAAGGGCATCACTTACAAACTC	(1)
23	GAPDH F	TTGATGGCAACAATCTCCAC	(3)
24	GAPDH R	CGTCCCGTAGACAAAATGGT	(3)
25	TNF-α F	TCTCATGCACCACCATCAAGGACT	(3)
26	TNF-α R	ACCACTCTCCCTTTGCAGAACTCA	(3)
27	C1q F	CTCAGGGATGGCTGGTGGCC	(3)
28	C1q R	CCTTTGAGACCCGGCCTCCCC	(3)
29	IL-1β F	CAACCAACAAGTGATATTCTCCATG	(3)
30	IL-1β R	GATCCACACTCTCCAGCTGCA	(3)
31	IL-6 F	GGTGACAACCACGGCCTTCCC	(3)
32	IL-6 R	TTAAGCCTCCGACTTGTGAAGTGGT	(3)
33	C3 F	CCAGCTCCCCATTAGCTCTG	(3)
34	C3 R	GCACTTGCCTCTTTAGGAAGTC	(3)

Forward and reverse primer sequences used in this study.

Data Set S1: Patient information from Johns Hopkins Cohort and Cleveland Clinic Cohort.

Data Set S2: Details of numerical p-values of all statistical tests that were performed.

SI References

1. X. Wang, A. Spandidos, H. Wang, B. Seed, PrimerBank: a PCR primer database for quantitative gene expression analysis, 2012 update. *Nucleic Acids Res* 40, D1144–1149 (2012).
2. P. T. Chen, Z. T. Chen, W. C. Hou, L. C. Yu, R. P. Chen, Polyhydroxycurcuminoids but not curcumin upregulate neprilysin and can be applied to the prevention of Alzheimer's disease. *Sci Rep* 6, 29760 (2016).
3. J. S. Park *et al.*, Blocking microglial activation of reactive astrocytes is neuroprotective in models of Alzheimer's disease. *Acta Neuropathol Commun* 9, 78 (2021).
